# Transcriptome-Mining for Single-Copy Nuclear Markers in Ferns

**DOI:** 10.1371/journal.pone.0076957

**Published:** 2013-10-08

**Authors:** Carl J. Rothfels, Anders Larsson, Fay-Wei Li, Erin M. Sigel, Layne Huiet, Dylan O. Burge, Markus Ruhsam, Sean W. Graham, Dennis W. Stevenson, Gane Ka-Shu Wong, Petra Korall, Kathleen M. Pryer

**Affiliations:** 1 Department of Biology, Duke University, Durham, North Carolina, United States of America; 2 Department of Zoology, University of British Columbia, Vancouver, British Columbia, Canada; 3 Systematic Biology, Department of Organismal Biology, Evolutionary Biology Centre, Uppsala University, Uppsala, Sweden; 4 Department of Botany, University of British Columbia, Vancouver, British Columbia, Canada; 5 Royal Botanic Garden Edinburgh, Edinburgh, Scotland; 6 New York Botanical Garden, Bronx, New York, United States of America; 7 Department of Medicine, University of Alberta, Edmonton, Alberta, Canada; 8 BGI-Shenzhen, Beishan Industrial Zone, Yantian District, Shenzhen, China; George Washington University, United States of America

## Abstract

**Background:**

Molecular phylogenetic investigations have revolutionized our understanding of the evolutionary history of ferns—the second-most species-rich major group of vascular plants, and the sister clade to seed plants. The general absence of genomic resources available for this important group of plants, however, has resulted in the strong dependence of these studies on plastid data; nuclear or mitochondrial data have been rarely used. In this study, we utilize transcriptome data to design primers for nuclear markers for use in studies of fern evolutionary biology, and demonstrate the utility of these markers across the largest order of ferns, the Polypodiales.

**Principal Findings:**

We present 20 novel single-copy nuclear regions, across 10 distinct protein-coding genes: *ApPEFP_C, cryptochrome 2, cryptochrome 4, DET1, gapCpSh*, *IBR3, pgiC, SQD1, TPLATE*, and *transducin*. These loci, individually and in combination, show strong resolving power across the Polypodiales phylogeny, and are readily amplified and sequenced from our genomic DNA test set (from 15 diploid Polypodiales species). For each region, we also present transcriptome alignments of the focal locus and related paralogs—curated broadly across ferns—that will allow researchers to develop their own primer sets for fern taxa outside of the Polypodiales. Analyses of sequence data generated from our genomic DNA test set reveal strong effects of partitioning schemes on support levels and, to a much lesser extent, on topology. A model partitioned by codon position is strongly favored, and analyses of the combined data yield a Polypodiales phylogeny that is well-supported and consistent with earlier studies of this group.

**Conclusions:**

The 20 single-copy regions presented here more than triple the single-copy nuclear regions available for use in ferns. They provide a much-needed opportunity to assess plastid-derived hypotheses of relationships within the ferns, and increase our capacity to explore aspects of fern evolution previously unavailable to scientific investigation.

## Introduction

Over the past twenty years, molecular phylogenetic approaches have radically altered our understanding of relationships in the fern tree of life. Arguably the most important finding (and among the most contentious) is that ferns, including the horsetails (*Equisetum*) and whisk ferns (Psilotaceae), form a clade sister to seed plants [1]. Ferns are therefore one of the great vascular plant radiations; only the angiosperm clade has more extant species. Broad studies of fern phylogeny, e.g., [1-18] have increasingly found stronger resolution and support across the majority of the backbone nodes, many of which were unanticipated based on morphological data. This rewriting of fern phylogeny has resulted in a novel emerging consensus of deep fern relationships [19-22]. Similarly, new molecular data have greatly facilitated inquiries into fern relationships at much finer scales, such as within genera, e.g., [23-46].

The vast majority of these studies, however, have been limited to data from the plastid genome; nuclear and mitochondrial data have not been widely used, cf. [4,9,47-49]. This dependence upon plastid data reflects a general absence of genomic resources available for ferns [50-53]—for example, no fern mitochondrial or nuclear genome has been sequenced yet—which has impeded the development of novel markers. To date only seven nuclear regions have been used in fern phylogeny investigations: ITS [13,54-57]; ribosomal 18S [4,58]; *LEAFY* [59-62]; *gapCpSh* (*gapCp “short”*) [36,42,43,49,57,63-67]; *gapCpLg* (*gapCp “long”*) [67]; *cam* [67]; and *pgiC* [57,59,66,68-75].

This strong reliance on the plastid genome makes fern phylogenetics vulnerable to misleading inferences, such as failures of this linkage group to track the organismal divergences (e.g., due to deep coalescence or reticulation). In addition, plastid data are poorly suited for species-level work in the many fern groups that have reticulate evolutionary histories [76-79]. Polyploidy and hybridization are common in ferns [80], and fully unraveling relationships in these groups will require the development of multiple unlinked markers. Regions from the nucleus are particularly attractive for this purpose because that genome has multiple linkage groups that are expected to have an elevated rate of evolution in ferns, e.g., [49].

The recent sequencing of approximately 1000 green plant transcriptomes by the One Thousand Plants Project (1KP; onekp.com) provides an unprecedented opportunity to facilitate the development of novel low-copy nuclear markers for use in ferns. There is no fern nuclear genome that has been sequenced to date, and only a handful of EST libraries, sequenced plastomes, or transcriptomes, e.g., [81-89]. Included in the 1KP sampling (as of January 2013, when we finished the sampling for this project) are 62 fern accessions, comprising 60 unique species. Our sampling from this time point is particularly rich in members of the leptosporangiate order Polypodiales, especially Pteridaceae (*sensu* [20,90]) and eupolypods II (*sensu* [8,16,19,91]), but also includes representatives of each of the major eusporangiate clades (Ophioglossales, Psilotales, Equisetales, Marattiales), as well as each of the leptosporangiate orders, except for the Osmundales [20]. Additional taxa, including representatives of the Osmundales, were sequenced in the 1KP project after we had finished our sampling. The full list—including algae, bryophytes, lycophytes, ferns, and seed plants—is available at http://www.onekp.com/samples/list.php.

Here, we utilize these transcriptome data to design primers for 20 nuclear markers across ten protein-coding genes. Our primary goals are: 1) to provide primers that will amplify single-copy nuclear markers across the majority of the Polypodiales; 2) to demonstrate the relative success of those primers in amplifying the desired region from genomic DNA, using a test set of 15 diploid Polypodiales species, and; 3) to characterize the resulting sequences and their efficacy in inferring relationships at various phylogenetic depths. In addition, we provide transcriptome alignments—curated broadly across ferns—for each of our target loci (including closely related paralogs in the case of gene families) to assist other investigators in designing primers for fern taxa of interest outside of the Polypodiales.

## Results

### Primer Development

**Figure 1:**
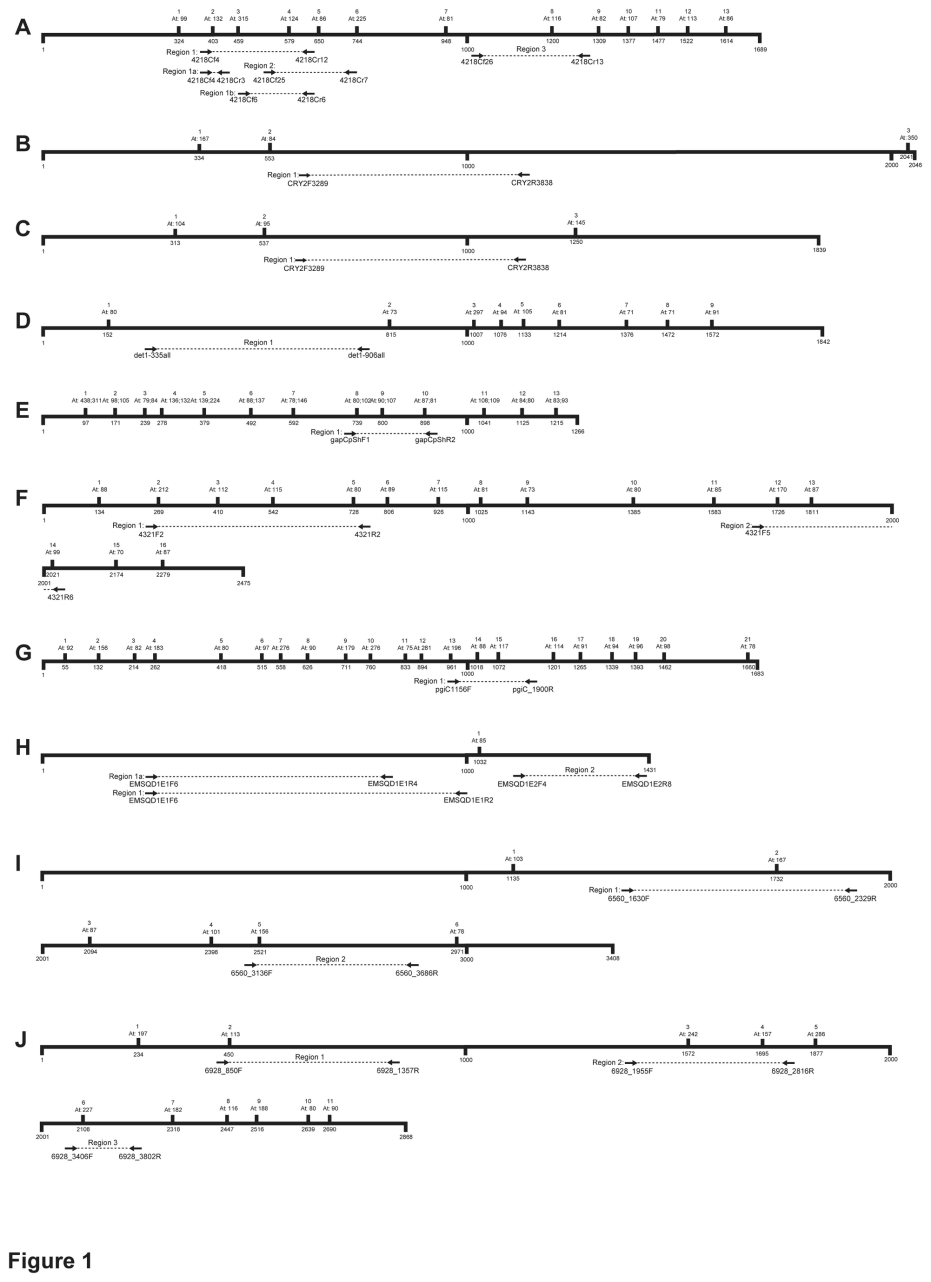
Schematic diagrams of the ten nuclear genes for which we developed fern-specific primers. (A) *ApPEFP_C*; (B) *CRY2*; (C) *CRY4*; (D) *DET1*; (E) *gapCpSh*; (F) *IBR3*; (G) *pgiC*; (H) *SQD1*; (I) *TPLATE*; (J) *transducin*. Each subset of the figure represents one protein-coding locus, using the most closely related *Arabidopsis thaliana* homolog as the template. The coding sequence is measured (in base pairs) along the bottom of the thickened horizontal line, with each locus wrapping onto a new line every 2000 base pairs, when necessary. Intron location, number, and length (in base pairs in *Arabidopsis*) are given above the line. Also shown below the line are the priming locations for each of the markers we developed. For *gapCpSh*, intron locations are based on *Arabidopsis* *gapCp1*: the first two exons of *Arabidopsis* *gapCp2* are each one codon shorter than in *gapCp1*.

**Figure 2:**
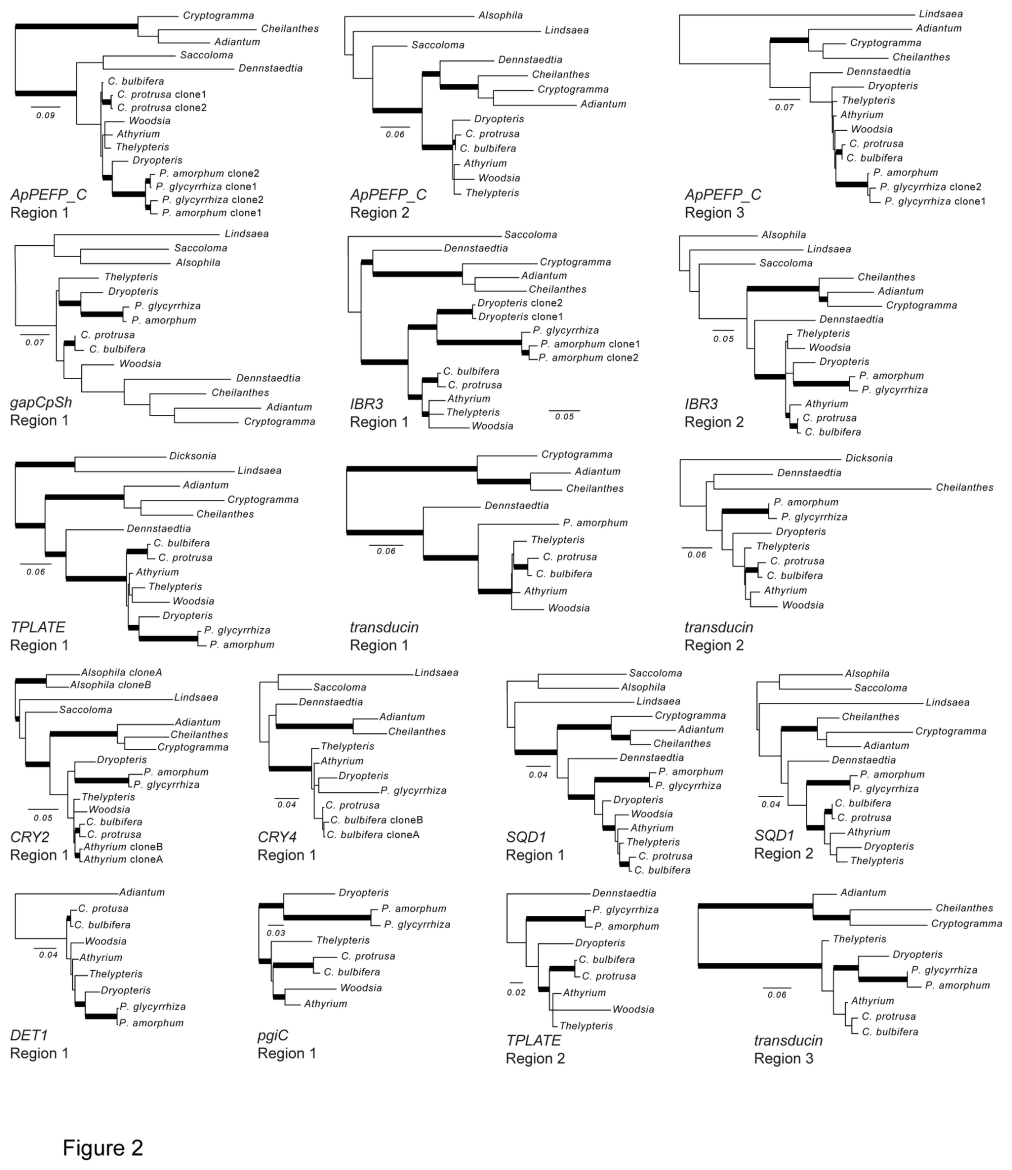
Maximum likelihood phylograms for each region, including only those taxa that were successfully sequenced from our 15-taxon genomic DNA test set. Bold branches indicate strong support (≥70% bootstrap support). Scale bars are in units of substitutions per site. In the taxon names, “*C*.” and “*P*.” refer to *Cystopteris* and *Polypodium*, respectively. These phylograms are unrooted, but oriented as if rooted by the Cyatheales (or our best guess, when the Cyatheales accession did not sequence successfully), when space permits.

We developed primer pairs for 20 regions across a total of ten distinct single-copy protein-coding genes: *ApPEFP_C, cryptochrome 2, cryptochrome 4, DET1, gapCpSh*, *IBR3, pgiC, SQD1, TPLATE*, and *transducin* (Tables 1, 2, 3; Figure 1). Each primer pair successfully amplifies the majority of taxa in our genomic DNA test set (comprising DNA from 15 diploid Polypodiales species; Table 3; Figure 2; Appendix S1). In general, we only attempted to sequence PCR products that had strong single bands (viewed with agarose gel electrophoresis). Many of the missing sequences are likely to be attainable by applying cloning protocols (e.g., see 64). Those sequences that we did attain by cloning are noted in the Methods section and in Table 3.

**Table 1:** Summary of the genes for which we designed primers.

			Length (CDS; in bp)		*Arabidopsis*		
	Abbreviation	Protein Name	*Arabid.*	Ferns	TAIR Gn#	# of Introns	Chromo. #
1	*ApPEFP_C*	appr-1-p processing enzyme family protein	1689	~1650-1743	AT1G69340	13	1
2	*CRY2*	cryptochrome 2	2046	~2000	AT4G08920	3	4
3	*CRY4*	cryptochrome 4	1839	~2100	AT1G04400	3	1
4	*DET1*	Nuclear-localized regulator of plant development	1632	~1600-2700	AT4G10180	9	4
5	*gapCpSh*	Plastid-localized GAPDH, short copy	1266; 1260	1315	AT1G79530; AT1G16300	13	1
6	*IBR3*	IBA-Response 3 (acyl-CoA dehydrogenase)	2475	~2445-2490	AT3G06810	16	3
7	*pgiC*	glucose-6-phosphate isomerase / sugar isomerase family protein	1683	--^a^	AT5G42740	21	5
8	*SQD1*	Sulfoquinovosyldiacylglyerol 1	1431	~1515-1521	AT4G33030	1	4
9	*TPLATE*	a cytokinesis protein targeted to the cell plate	3531	--^a^	AT3G01780	6	3
10	*transducin*	transducin family protein / WD-40 repeat family protein	2868	--^a^	AT3G21540	11	3

For each gene, we list its length in ferns and in *Arabidopsis*, provide the TAIR accession number for the *Arabidopsis* sequence (as well as its number of introns and chromosomal position). The TreeBASE accession number for our “all-in” fern alignments is S14616. Comparisons with *Arabidopsis thaliana* are based on the most closely related homolog(s). ^a^ These loci were trimmed to a focal region prior to completion, so the full length of the coding DNA sequence (CDS) is unknown.

**Table 2:** Priming details for 20 novel nuclear markers.

		**Primers (Forward, Reverse)**		
**Protein Region**		**Name**	**Sequence (5'–3')**	**PCR Program**
*ApPEFP_C*	1	4218Cf4, 4218Cr12	GGACCTGGSCTYGCTGARGAGTG, GCAACRTGAGCAGCYGGTTCRCGRGG	6512035
*ApPEFP_C*	1a	4218Cf4, 4218Cr3	GGACCTGGSCTYGCTGARGAGTG, TCGTAAGCRTTYGTTACTTTDGCC	5506035
*ApPEFP_C*	1b	4218Cf6, 4218Cr6	AAAGTTATACATACTGTTGGTCC, GCAACATGAGCAGCTGGTTCACGAGG	5506035
*ApPEFP_C*	2	4218f25, 4218r7	AATGCTCTRAGTCAYTGYTAYMGATC, TTGTAAATCTCTGTRTCRGATGYYGT	5509035
*ApPEFP_C*	3	4218f26, 4218r13	CAAAGGCGCAARGAACARTGGGARAGRGTTGC, TCAAGACAYCGTAGCAGRAARTGBGCYCC	6512035
*CRY2*	1	CRY2F3289_Pt, CRY2R3838_Pt	AGGATGARYTGGAGAAAGGYAGCAATG, GTRTCCCAGAAATAYTTCATACCCC	5209035
*CRY4*	1	CRY2F3289_Pt, CRY2R3838_Pt	AGGATGARYTGGAGAAAGGYAGCAATG, GTRTCCCAGAAATAYTTCATACCCC	5209035
*DET1*	1	det1-335all, det1-906all	TATGAYGTGGARTGCCCAGAT, TCTCTGCAGAAHKGYCCAA	5506035
*gapCpSh*	1	gapCpShF1, gapCpShR2	TGCACMACHAACTGCCTTGCRCCBCTTGC, CCATTYARCTCTGGRAGCACCTTTCC	6512035
*IBR3*	1	4321F2, 4321R2	TCTGCMCATGCMATTGAAAGAGAG, CCCARKGTYGAAAGYTCCCAATC	6312035
*IBR3*	2	4321F5, 4321R6	ATGACYGAACCAGATGTKGCDTCVTCRGATGC, TGRTGGAGYCTKCCTGGGCCTA	6512035
*pgiC*	1	pgic_1156F, pgiC_1900R	GGYCTYYTRAGYGTYTGGAATGT, GGTGAAATYGAYTTYGGDGARC	5812035
*SQD1*	1	EMSQD1E1F6, EMSQD1E1R2	GCAAGGGTACHAAGGTHATGATCATAGG, CCTTTDCCRTARACTGTAAGAGGATG	5512035
*SQD1*	1a	EMSQD1E1F6, EMSQD1E1R4	GCAAGGGTACHAAGGTHATGATCATAGG, GCGTGARTCRTGCACTTTGCTRAGATG	5512035
*SQD1*	2	EMSQD1E2F4, EMSQD1E2R8	CGHGTRTTYAATCARTTYACAGAAC, GTCACTGTHACAGGTTTYACDCCAGC	5512035
*TPLATE*	1	6560_1630F, 6560_2329R	TGCYTAGTSGARAGYTGYTTTCA, AATGTAGCAACTAACAGGCTTCAAGA	5812035
*TPLATE*	2	6560_3136F, 6560_3686R	AAYCTYCARCATCTYCAGTCTC, GCAACKGCHGCDGTBGAAAG	5812035
*transducin*	1	6928_850F, 6928_1357R	TTRCGBGGRCAYARAGATCA, GGAWCSTTARTSGGYTGCCAA	5812035
*transducin*	2	6928_1955F, 6928_2816R	AAGGCDGGRAARCTNGAGAT, ATGGAYATYTCCWCYGATGC	5812035
*transducin*	3	6928_3406F, 6928_3802R	TCBATTCGRMGATGGGAGCG, CAAACYCARGARWCYSTGAC	5812035

The first two digits of the PCR program is the annealing temperature, followed by a three-digit elongation time (in seconds), followed by the number of cycles.

**Table 3:** Sequence characteristics for the single-copy regions developed in this study.

		**# of Differences**		**Length of Amplified Region**														
**Protein Region**		**Cys. pair**	**Poly. pair**	**Cya.**	**Lin.**	**Sac.**	**Adi.**	**Che.**	**Cry.**	**Den.**	**Dry.**	**P.am.**	**P.gl.**	**Ath.**	**C.bu.**	**C.pr.**	**The.**	**Woo.**
*ApPEFP_C*	1	>38; >25^a^	50; 25^b^	?	?	>751	>690	>829	721	931	761	846; >871^c^	843; 837^c^	>849	>866	932; 934^c^	687	939
*ApPEFP_C*	1a	>13; >12^a^	10; 9^b^	145	?	258^d^	321^d^	281	300^d^	293	267	~257	~255	201^d^	245	161	>225	255
*ApPEFP_C*	1b	6; 6^a^	11; 3^b^	?	?	?	224	209^d^	223^d^	269	223	224; 223^d^	216; 223^d^	224	214^d^	223^d^	223	222
*ApPEFP_C*	2	5	?	382	334	360	360	340	357	406	359	?	?	360	359	358	359	357
*ApPEFP_C*	3	14	11; 25	?	377	?	>506	>776	770	802	454	385	378; 384^c^	490	482	461^c^	489	457
*CRY2*	1	9	14	516	516	516	516	516	516	?	516	516	516	516	516	516	516	516
*CRY4*	1	2	?	?	516	516	516	516	?	516	516	?	516	516	516	516	516	?
*DET1*	1	~5	6	?	?	?	~630	?	?	?	667	668	668	~670	665	664	669	669
*gapCpSh*	1	?	14	?	455	459	?	?	482	476	522^c^	531	525	?	?	466	517	592
*IBR3*	1	>16	29; 31	?	?	870	817	>700	827	836	819; 828^c^	843^c^	844	815	>819	840	>910	821
*IBR3*	2	~6	~21	~600	>766	611	~574	581	568	~1196	?	~590	595	586	~580	~582	579	588
*pgiC*	1	>32	>19	674	?	?	?	?	?	?	625	>615	678	>474	>664	>581	619	620
*SQD1*	1	10	>8^e^	?	700	668	700	700	700	700	700	?	700	700	700	700	700	685
*SQD1*	1a	8	8	530	530^f^	530^f^	530^f^	530^f^	530^f^	530^f^	530^f^	529	530^f^	530^f^	530^f^	530^f^	530^f^	530^f^
*SQD1*	2	1	3	264	263	264	264	256	264	263	264	264	264	264	264	233	264	?
*TPLATE*	1	12	14	719	>657	?	646	>561	>529	627	711	696	698	>638	710	>662	>692	687
*TPLATE*	2	5	10	>327	?	?	?	551	?	529	512	497	493	302	427	424	722	541
*transducin*	1	11	?	?	?	?	402	397	426	416	?	>381	?	436	437	435	420	421
*transducin*	2	11	>7	>521	?	?	?	>426	?	539	534	529	>445	518	525	517	514	504
*transducin*	3	6	7	?	?	?	227	308	231	?	268	251	251	242	244	244	243	242

Cys: *Cystopteris*, Poly: *Polypodium*, Cya: Cyatheales, Lin.: *Lindsaea*, Sac.: *Saccoloma*, Adi: *Adiantum*, Che: *Cheilanthes*, Cry: *Cryptogramma*, Den: *Dennstaedtia*, P.am.: *Polypodium amorphum*, P.gly.: *Polypodium glycyrrhiza*, Ath.: *Athyrium*, C.bu.: *Cystopteris bulbifera*, C.pr.: *Cystopteris protrusa*, The: *Thelypteris*, Woo: *Woodsia*. ^a^ The two values come from comparing the single incomplete *Cystopteris bulbifera* sequence against two sequences cloned from *C. protrusa*. ^b^ This locus has a duplication in *Polypodium*; these values are the number of bp changes between each of the ortholog pairs. ^c^ Required cloning. ^d^ These lengths are derived from the corresponding portion of the *APPEFP_C* Region 1 alignment (we did not attempt to amplify Region 1a or 1b for all taxa). ^e^ For *P. amorphum* we were not able to amplify Region 1 for this locus, only Region 1a. ^f^ These lengths are derived from the corresponding portion of the *SQD1* Region 1a alignment (Region 1a for all taxa).

#### ApPEFP_C

We developed primers for three regions of *ApPEFP_C* (Figure 1A), and for one of those regions (Region 1) we designed non-overlapping internal primers that can amplify two smaller subset regions (Figure 1A). Region 1 is approximately 700–1000bp long in ferns (Table 3) and spans introns 2, 3, and 4, exons 3 and 4, and half of exon 5 (Figure 1A). It could be direct-sequenced for most taxa in our genomic DNA test set, although cloning was necessary for *Polypodium* (due to a hypothesized gene duplication in the Polypodiaceae; see Figure 2) and *Cystopteris protrusa*. Within Region 1, the additional reverse primer 4218Cr3 allows for the amplification of the subset Region 1a, and the forward primer 4218Cf6 yields Region 1b (Figure 1A). Both these smaller regions are approximately 200–300 bp long. Region 1a is the more variable of the two, whereas Region 1b has good length conservation among taxa and is easy to align across the complete breadth of the Polypodiales (Table 3). Region 2 overlaps with the 3’ end of Region 1; it includes a portion of exon 4, introns 4 and 5, exon 5, and most of exon 6 (Figure 1A). Finally, Region 3 is intermediate in length and includes much of the large exon 8, intron 8, and most of exon 9. In the eupolypods, Region 3 ranges in length from approximately 400 to 500 bp, but is consistently larger in the Pteridaceae and Dennstaedtiaceae (reaching 802 bp in *Dennstaedtia*). It amplified and direct-sequenced well (Figure 2), but required cloning for *P. glycyrrhiza* and *C. protrusa* (see above); however, the *P. amorphum* Region 3 sequence was clean (not double-peaked when directly sequenced), and did not require cloning.

#### CRY2 and CRY4

We designed primers to target a 516 bp region in the third exon of *CRY2* (Figure 1B). However, these primers also cross-amplify the same region in *CRY4* (also 516 bp; Figure 1C), and, less frequently, in *CRY3* and *CRY5* (see Figure S2). The PCR products thus cannot be directly sequenced. Nevertheless, after cloning, we recovered *CRY2* and *CRY4* for 14 and 11 (respectively) of the 15 taxa in our genomic DNA test set. These two loci are sufficiently divergent that assigning sequences to the correct copy is straightforward. *CRY2* appears to have higher sequence variation, with nine nucleotide differences between the *Cystopteris* species pair, whereas there are only two nucleotide differences between the same species pair in *CRY4* (*Cystopteris protrusa* and *C. bulbifera* constituted one of the two pairs of closely related species that we used as a metric for informativeness at shallow phylogenetic depths—see Methods and Table 3).

#### DET1

We focused on a single region of *DET1* (Figure 1D). The primer pair 4321F2-4321R2 amplifies a ~670 bp region that includes most of the second exon (in *Arabidopsis*; ferns contain an additional intron within this region). All sequences were obtained by direct-sequencing.

#### GapCpSh

We designed primers for a single region of *gapCpSh*. The forward primer is situated just before intron 8 and the reverse priming site is just after intron 10 (Figure 1E); this region ranges in length from ~450 to 590 bp in our genomic DNA test set. In general *GapCpSh* amplified and direct-sequenced well, although we were not able to obtain clean sequences for *Alsophila, Adiantum, Cheilanthes, Athyrium*, or *Cystopteris bulbifera* (cloning not attempted) and the *Dryopteris* sequence required cloning. This region physically overlaps with the *gapCp* region amplified with the primers of Schuettpelz et al. [64], but differs in that our primers are specific to the *gapCp Short* (*sensu* [64]) copy in Polypodiales, and amplify a region slightly shorter than that of Schuettpelz et al. [64].

#### IBR3

We designed primers for two regions of *IBR3*. Region 1 spans introns 2–5 and exons 3–5 (Figure 1F); it is approximately 900bp long in the Polypodiales species we examined (Table 3). Region 2, at the 3’ end of the gene, is shorter, at around 600 bp in most species; however, it is much larger (1200 bp) in *Dennstaedtia*. It spans introns 12–14, exons 13 and 14, and the end of exon 12. Both regions amplified well, and gave clean direct sequences for the majority of taxa in our test set.

#### PgiC

We developed one novel primer pair for *pgiC*—a locus already known to work well in fern phylogenetics [57,66,71-75,92]. Our primers are situated in exons 14 and 16, amplifying introns 14, 15, and exon 15 (Figure 1G). The amplified region ranges in length between 600 and 700bp across our test set (Table 3), and the range of variation observed is appropriate for resolving infrageneric relationships (Table 3 and see citations above). It amplified and direct-sequenced well for 10 of the 15 test set taxa; *Lindsaea, Saccoloma, Dennstaedtia, Cheilanthes* and *Adiantum* failed amplify and/or direct-sequence cleanly.

#### SQD1

Primers were designed for two regions of *SQD1*: a 700 bp region within the first exon and a 264 bp region within the second exon (Figure 1H; Table 3). The Region 1 forward and reverse primers—EMSQDE1F6 and EMSQDE1R2—produced successful amplifications for 13 of the 15 taxa in our test set. An additional reverse primer, EMSQDE1R4, was designed to amplify a 530 bp subset (henceforth designated Region 1a; Figure 1H, Table 3), which resulted in successful amplification and direct-sequencing of the two remaining accessions (*Alsophila* and *Polypodium amorphum*). Primers designed for Region 2 resulted in the successful sequencing of all taxa in our test set, except *Woodsia*.

#### TPLATE

We designed primers for two regions of *TPLATE* (Figure 1I). Our primers for Region 1 were highly successful (only *Saccoloma* failed to amplify). It spans part of exon 2, all of intron 2, and part of exon 3, ranging in length among taxa in our test set from 650-720bp. Region 1 had moderate levels of variation (16 differences for the *Cystopteris* species pair, and 15 for *Polypodium*; Table 3). Region 2 is 400-550bp long (Table 3), and slightly less variable than Region 1. Its primers are situated in exon 5 and exon 6 and span intron 5 (Figure 1I). We managed to sequence this region for 11 of the 15 test set taxa (*Adiantum, Cryptogramma, Lindsaea*, and *Saccoloma* were unsuccessful). In our phyogenetic analyses of these data, we had to exclude *Cheilanthes* and *Dicksonia* because they were too divergent from the other taxa to align confidently.

#### Transducin

For *transducin* we designed primers for three regions. Region 1 extends from the 5’ end of intron 2 through to the middle of exon 3 (Figure 1J), and is approximately 400-450 bp long (Table 3). Amplification and sequencing was successful for 10 of our 15 test set species (*Lindsaea, Saccoloma, Dicksonia, Dryopteris*, and *Polypodium glycyrrhiza* were unsuccessful). Region 2 is approximately 550bp long, spanning exons 3 to 5 (Figure 1J, Table 3). It was amplified and sequenced successfully for 11 of the test set species (*Adiantum, Cryptogramma, Lindsaea*, and *Saccoloma* failed). Region 3 is 250-300 bp long, amplifying intron 6 and portions of exons 6 and 7 (Figure 1J, Table 3). It was successfully amplified and sequenced for all test set taxa except *Lindsaea* and *Saccoloma*.

### Model selection and the Polypodiales phylogeny

The combined alignment of our 19 newly developed regions (*SQD1* Region 1 and Region 1a were merged for these analyses) across our 15-taxon Polypodiales genomic DNA test set (the set of genomic DNAs that we used to test our new primer sets) is 9007 base pairs long; 42 percent of the sites are variable. Twenty-eight percent of the characters in this alignment are missing (i.e., gaps or question marks). We investigated five models for these data (where “model” refers to the product of the partitioning scheme and the substitution model applied to each subset of the data), which ranged from one subset and 37 free parameters to 30 subsets and 238 free parameters (see Methods; Table 4; Appendix S2). In their extremes, these models differed by over 2600 in their log likelihood scores, and by nearly 4500 Bayesian information criterion (BIC) points (Table 4). Model selection based on the BIC favored a relatively simple model for these data: four data subsets corresponding to the three codon positions and the noncoding sites, respectively, with the first two codon positions optimized under a GTR+G model and the two other subsets including an additional proportion invariant parameter (GTR+I+G; Table 4; Appendix S2). No parameters, other than relative branch lengths, were linked across partitions.

**Table 4:** Model comparison, by the Bayesian Information Criterion (BIC).

					Support Values by Branch (ML bootstrap percent)											
Model	lnL	BIC	Subsets	Param.	A	B	C	D	E	F	G	H	I	J	K	L
1: Pos. & Locus	-40574.4	83316.0	30	238	56	**100**	53	**100**	**100**	**100**	**100**	**99**	**100**	**100**	**87**	58
2a: Locus (each)	-42767.5	86819.0	19	141	49	**100**	**74**	**100**	**100**	**100**	**100**	**96**	**100**	**100**	**88**	61
2b: Locus (scheme)	-42748.0	86470.3	11	107	52	**100**	**72**	**100**	**100**	**100**	**100**	**97**	**100**	**100**	**87**	67
3: Pos.	-40875.4	82370.0	4	68	55	**100**	41	**100**	**100**	**100**	**100**	**98**	**100**	**100**	**82**	42
4: Unpartitioned	-43190.2	86717.3	1	37	66	**100**	61	**100**	**100**	**100**	**100**	**95**	**100**	**100**	**80**	41

**Figure 3:**
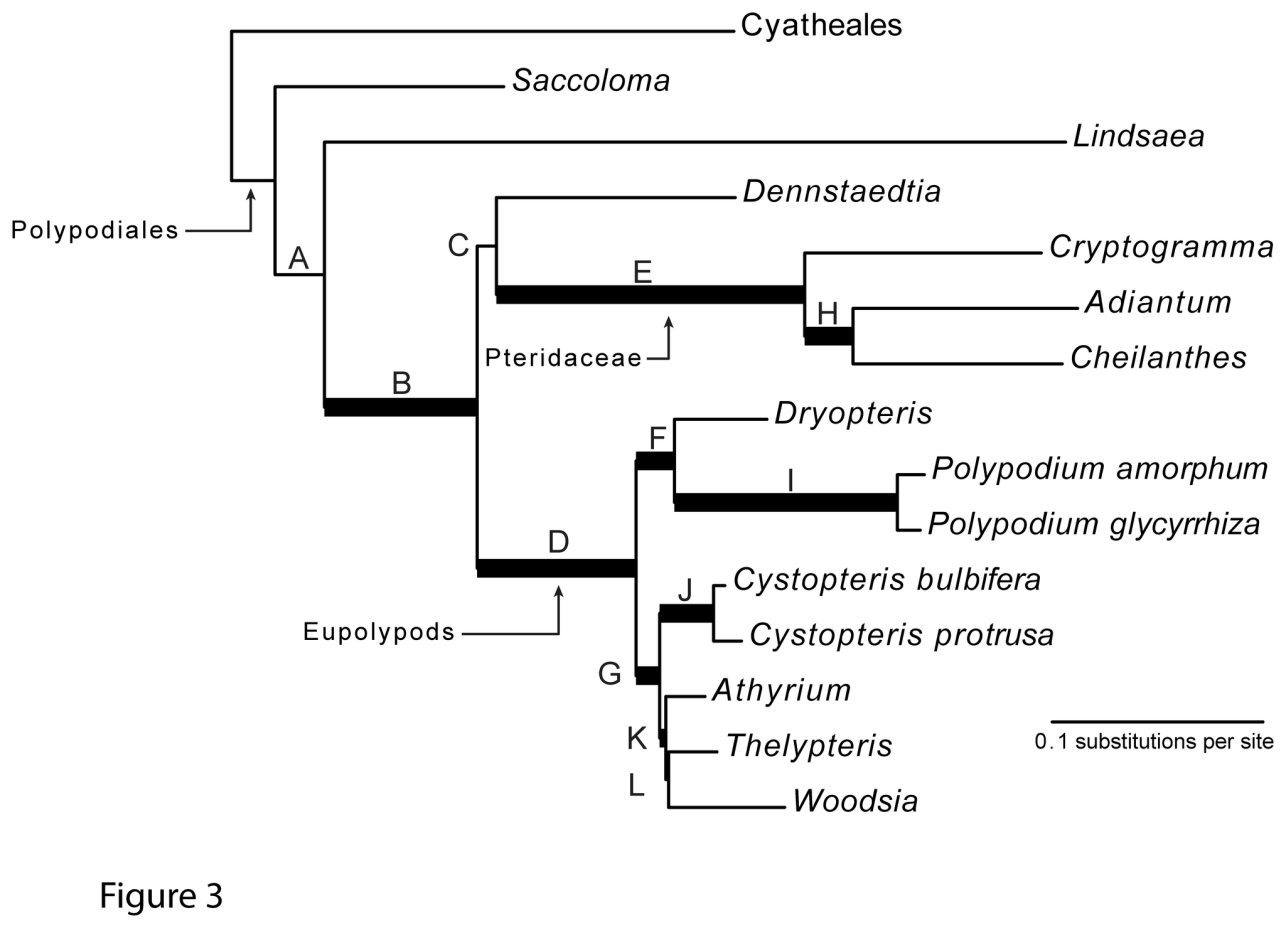
Combined data maximum likelihood phylogram of our 15-taxon genomic DNA test set. Analyses were performed under our best-fitting model (model 3, see Table 3). Bold branches indicate strong support (≥70% bootstrap support); internal branches are labeled A – L for ease of discussion.

Values in bold face indicate strong support (≥70%). Branch designations (A – L) refer to Figure 3. Model 1 is the best *PartitionFinder* scheme given each codon position, for each locus, as the data blocks. In model 2a each locus gets its own partition, across codon positions. Model 2b is the best *PartitionFinder* scheme given the loci as the data blocks. Model 3 is partitioned by codon position, across loci. Model 4 is not partitioned. For substitution model parameterization, see Appendix S2. Subsets = the final number of subsets (“partitions”) for that model. Param. = number of free parameters.

Model parameterization had strong effects on the fit to our data and on our subsequent inference. In general, the models without a codon-position component to their partitioning schemes (the unpartitioned model—model 4, and the two models partitioned by locus—models 2a and 2b) performed very poorly. The addition of codon-position-based partitions dramatically improved model fit (Table 4), such that the subsequent addition of locus-based partitions resulted in a decline in model fit. For example, the BIC favored the simple by-position partitioning scheme (model 3: four subsets, 68 free parameters) over the best by-position-and-locus scheme (model 1: 30 subsets, 238 free parameters; Table 4).

Model choice impacted the ML estimate of topology, but only slightly: model 2a resolved *Lindsaea* as sister to the rest of the Polypodiales, whereas all other models put *Saccoloma* in that position. However, model choice had a stronger effect on support values. The most extreme example of this effect was branch C (Figure 3), which ranged from 41 percent ML bootstrap support under model 3 (our best-fitting model) to 74 percent support under model 2a (our worst-fitting model; Table 4).

Despite the high proportion of missing data, ML analyses of this alignment under our best-fitting model yielded a well supported phylogeny, with only three branches lacking strong support: Branch A (the earliest divergence in the Polypodiales), Branch C (the position of *Dennstaedtia* with respect to Pteridaceae and the eupolypods), and Branch L (the relationships among the three non-Cystopteridaceae eupolypod II accessions; branch labels refer to Figure 3). The transcriptome alignments themselves also contain rich phylogenetic data. The analysis of these data is beyond the scope of this paper, and is the focus of a forthcoming manuscript.

## Discussion

### Single-copy locus identification, and alignment inference

Our approach to single-copy locus identification and development was highly effective, albeit labor-intensive. Specifically, we combined repeated rounds of sequence-merging and tree-building for each of our candidate loci. This approach allowed us to build compact matrices (long reads for each accession with relatively little missing data) despite the fragmentary nature of the source assemblies. Our hands-on method of alignment development and curation also allowed us to identify novel gene duplication events, to distinguish among paralogs, and to detect both contaminants and misidentifications. We are therefore confident that our final alignments are both of high data quality (data density, taxon representation, and alignment inference quality) and high accuracy (free from contaminants, inter-paralog chimaeras, etc.; see Figures S1-S9). This approach was only possible given the modest amount of data that we worked with (the “moderate data approach”, see 6), and would not scale to large genomic datasets [93,94].

### Nuclear genes with newly designed primer sets

***ApPEFP_C*** is poorly characterized and does not appear to have a history as a phylogenetic marker; in *Arabidopsis* it is described simply as an “appr-1-p processing enzyme family protein” [95]. *ApPEFP*, formerly thought to be single-copy across much of the green plants (1KP data, Norman Wicket, pers. comm.), appears to have duplicated early within leptosporangiate ferns (Figure S1). We designated the pre-duplication version *ApPEFP_A* and the two post-duplication copies *ApPEFP_B* and *ApPEFP_C*, respectively. The *ApPEFP*_*B*/*C* duplication may have taken place early within leptosporangiate diversification; *Dipteris* and *Lygodium* each have both copies. Further sampling (particularly of the Osmundales) will be necessary to refine the timing of this duplication.

There appears to be an additional duplication of *ApPEFP_A* in the Equisetales, and probably at least one other duplication in the Marattiales (Figure S1). The *ApPEFP_B* phylogeny is well resolved, with no additional apparent duplications in this paralog. *ApPEFP_C* was the best represented in our transcriptome sampling, and is the only copy that we pursued for primer generation. Within *ApPEFP_C* there is an apparent duplication in the Polypodiaceae. This duplication occurred in the ancestry of *Polypodium*, after the divergence of *Phlebodium* and *Pleopeltis* (Figure S1).

***CRY2*** and ***CRY4*** are members of the cryptochrome family of blue light photoreceptors, a gene family known in both prokaryotes and eukaryotes. In *Arabidopsis*, cryptochromes are responsible for circadian clock entrainment, flower induction, and de-etiolation [96,97]. Their function in ferns is not entirely clear, although some copies may be involved in inhibition of spore germination under blue light [98]. There are three cryptochrome copies in *Arabidopsis* [97], and five copies described in *Adiantum capillus-veneris* [98]. In our data, we recover these five copies (which we denote as *CRY1* through *CRY5*) from the majority of the polypod fern transcriptomes (Figure S2). The gene family appears to have evolved via an initial duplication on the fern stem lineage, producing the ancestral *CRY1*/*2* and *CRY3*/*4* paralogs. CRY5 originated around this time, too, perhaps from duplication of the CRY1/2 paralog. Two additional duplications followed, producing *CRY1* and *CRY2* on the stem branch of Cyatheales + Polypodiales, and *CRY3* and *CRY4* on the stem branch of Polypodiales, after the divergence of Cyatheales (Figure S2).

The first intron of fern *CRY2* is currently being developed as a phylogenetic marker in *Deparia* (Li-Yaung Kuo pers. comm.) and *Adiantum* (Wanyu Zhang pers. comm.). We designed our primer pair to target the third exon instead, and found that it recovers the corresponding region from both *CRY2* and *CRY4* for most of our test set.

The ***DET1*** protein is an important regulator in the ubiquitin-proteasome system as part of the CDD (COP10-DET1-DDB1) complex. It also has been found to be a transcriptional co-repressor recruited to target genes by specific transcription factors [99]. The gene appears to be single copy in polypod ferns (Figure S3) and is present in other eukaryotes, including humans [100]. Of all the nuclear regions for which we designed primers, DET1 is the most conserved (Table 3).

***GapCpSh*** is a member of the glyceraldehyde-3-phosphate dehydrogenase (GAPDH) gene family and is one of the most frequently used nuclear loci in ferns, following the pioneering work of Ebihara et al. [63] and Schuettpelz et al. [64]. Land plants have four deeply divergent GAPDH genes—*gapA, gapB, gapC*, and *gapCp*—each of which is nuclear encoded. The first two are originally of mitochondrial origin, and the latter two were plastid encoded prior to their relocation to the nucleus [101-104]. Although we used only fern *gapCp* sequences as queries to build our all-in transcriptome alignments, our pool of transcriptome hits included representatives of each of the four main copies, as well as a fifth clade of uncertain identity (Figure S4). This myster copy appears to be a member of the GAPDH family (it is readily alignable to other members of the family and all our sequences in this clade have well-characterized members of the GAPDH family as their closest blast hits), but is deeply divergent from the known copies. It appears to be most closely related to the *gapC/gapCp* copies, but diverged from their ancestor prior to the *gapC*/*gapCp* duplication event (Figure S4a). Our transcriptome hits included a good representation of this mystery copy from across the Polypodiales, with an additional hit in *Anemia* (Schizaeales). Presumably it has been either lost from other ferns or was transcribed at insufficient levels to be captured in many of our source transcriptomes.

*GapA* and *gapB* are very poorly represented in our blast hits, as might be expected, given their phylogenetic distance from our query sequences. *GapC* sequences, however, are well represented, with a broad sample of sequences from the Ophioglossales and Polypodiales, and sparser representation from the Cyatheales (Culcita) and Salviniales (*Azolla* and *Pilularia*). Within the *gapC* portion of the phylogeny (Figure S4a), species are generally in their expected phylogenetic position, with two main exceptions. The first is the position of *Culcita* (a member of the Cyatheales) within the Pteridaceae (in the Polypodiales). This *Culcita* sequence, however, is very short (128 bp) and its position is likely an artifact due to limited data. The second irregularity is more difficult to explain: a clade of three Pteridaceae sequences is effectively sister to the rest of the leptosporangiate sequences and far from the Pteridaceae. The relationship among these three sequences corresponds with their expected species relationships, and each of the three species also has a “good” *gapC* sequence in the appropriate phylogenetic position. These three anomalous sequences may represent an otherwise uncaptured *gapC* duplication early in the leptosporangiate fern evolution.

The position of the Equisetales sequences is also ambiguous. Each of the two *Equisetum* accessions (*E. diffusum* and *E. hyemale*) has multiple *gapC/Cp* type sequences, but they fall together in a clade that is resolved in our maximum likelihood (ML) analyses as sister to the fern + seed plant *gapCp* clade, rather than in separate *gapC* and *gapCp* clades (Figure S4). Based on this result, we tentatively treat them as *gapCp* copies, with an *Equisetum*-specific *gapCp* duplication. Consistent with the results of Schuettpelz et al. [64], we recovered three main *gapCp* copy types in the ferns: a pre-duplication copy, and a duplication in the leptosporangiates forming *gapCp* “*short*” (*gapCpSh*) and *gapCp* “*long*” (*gapCpLg*). Schuettpelz et al. [64] hypothesized that the *gapCpSh*/*Lg* duplication event occurred near the base of the Polypodiales, or possibly more deeply (with subsequent losses, based on their sampling). Our transcriptome data suggest that the duplication very likely occurred at a point after the divergence of the Hymenophyllales, Gleicheniales, and Schizaeales, but prior to the divergence of the Salviniales, from the remaining leptosporangiates (Figure S4). Within the *gapCp* clade there is one group of sequences that is difficult to reconcile with the organismal phylogeny: a clade of five Cyatheales sequences (three from *Thyrsopteris*, and one each from *Plagiogyria* and *Culcita*) that appear to have diverged before the *gapCpSh*/*Lg* duplication (Figure S4b). The three species represented also have “good” *gapCpSh* and *gapCpLg* sequences, so it is unclear what paralog this anomalous clade represents.

*GapCpLg* is represented in our transcriptome sample by a single Salviniales sequence (*Pilularia*), and by sequences from the majority of our sampled species of Cyatheales and Polypodiales. The phylogeny of these sequences is consistent with the currently accepted fern topology [8,20], and does not show any indication of subsequent duplication. *GapCpSh* is even better represented, with sequences from both *Pilularia* and *Azolla*, plus broad representation across Cyatheales and Polypodiales. As with the *Adiantum*-specific *gapCpSh* duplication found by Rothfels and Schuettpelz [49], the two *Astrolepis*-specific duplications found by Beck et al. [42], and the *gapCp* duplication documented in the evolution of *Arabidopsis* [105] our data suggest at least two more duplications of *gapCpSh*: one in a common ancestor of *Culcita* and *Plagiogyria*, and another in the Lindsaeaceae (Figure S4b).

***IBR3*** has not been previously used as phylogenetic marker. It is related to acyl-CoA dehydrogenases and, while its subcellular location has not been confirmed, it contains a peroxisomal targeting sequence and likely is localized to that organelle [95,106]. *IBR3* appears to be present as a single copy throughout the fern tree, and is thought to be single copy across land plants (1KP data; Norman Wicket, pers. comm.). One possible exception in our data is in the Psilotaceae, where there may be a duplication (Figure S5).

***PgiC*** is one of the most extensively used nuclear markers in ferns (e.g., [57,59,66,71-75]). It also has a history in angiosperm phylogenetics, e.g., [107,108], was one of the most frequently used enzymes in allozyme studies, e.g., [109,110], and is single-copy in ferns [71] (Figure S6). The gene codes for phosphoglucose isomerase, an enzyme active in the glycolysis of glucose-6-phosphate isomerase [95]. In our phylogenetic analyses, we excluded the *Dicksonia* sequence because it was too divergent from the other taxa to align confidently.

The ***SQD1*** gene encodes a protein required for synthesis of sulfoquinovosyldiacylglycerol (SQDG), a well-characterized sulfolipid found in chloroplast membranes, and is widely distributed across land plants, green algae, and cyanobacteria [111]. It is hypothesized that *SQD1* permits proper functioning of photosystem II under phosphorous limited conditions [112]. Studies utilizing Southern hybridization demonstrated that *SQD1* is single-copy in *Arabidopsis thaliana* and the chlorophyte algae *Chlamydomonas reinhardtii* [113,114]. In silico analysis of fully annotated genomes indicated that *SQD1* is also present as a single copy in *Oryza sativa* and *Populus trichocarpa*, prompting the development of angiosperm-specific primers [115,116]. Additional genomic analyses confirmed single copies of *SQD1* in *Physcomitrella patens, Selaginella moellendorffii, Vitis vinifera, Zea mays*, and *Sorghum bicolor* [117]. Our study suggests that *SQD1* is a single copy gene for the majority of fern taxa (Figure S7). A notable exception is the presence of an apparent duplication in an ancestor of the Marattiaceae. Several other more-recent duplications have occurred in isolated genera or species such as *Lindsaea, Culcita macrocarpa* and *Nephrolepis exaltata*. Notably, our *Ophioglossum* (Ophioglossales) *SQD1* sequence is resolved as sister to *Lygodium* (Schizeaceae), a position incompatible with the current, accepted understanding of fern phylogeny [20]. It is possible that this is an alignment artifact. We do not suspect contamination, because an *Ophioglossum + Lygodium* clade is not recovered in phylogenies of any of other loci in this study.

***TPLATE*** has been identified as a cytokinesis protein involved in the formation of the cell plate [95,118]. Van Damme et al. [119] have also shown that it is important for the formation of viable pollen. It is a member of the group of putatively single-copy markers identified by the 1KP project (Norman Wicket pers. comm.), and to our knowledge has not previously been used as a phylogenetic marker. It is single-copy in our transcriptome sample except for possible duplications in the Ophioglossaceae (Figure S8).

The function of the ***transducin*** protein in plants is not well described. It belongs to the G-protein complex, which is involved in signaling across the cell membrane [120]. In *Arabidopsis* this complex is thought to be involved in the export and import of mRNA and protein to the nucleus [95]. It is a member of the group of putatively single-copy markers identified by the 1KP project (Norman Wicket pers. comm.), and is single-copy in our sample (Figure S9). To our knowledge it has not previously been used as a phylogenetic marker.

### Model selection and the Polypodiales phylogeny

The strong improvement in fit to our data that is provided by selecting a model with codon-position based partitions, and the correspondingly weak (or negative) contribution of locus-based partitions, is consistent with other studies [121-124]. This result both emphasizes the importance of including codon position information in model selection procedures, and suggests that our loci share organismal histories: the absence of strong by-locus effects on model fit suggests congruence among the gene trees. Also notable is the strong effect of model choice on ML bootstrap support levels (Table 4). Each of our five models was the best of its “class,” in the sense that each represented the optimal parameterization for the chosen partitioning scheme (by the Akaike information criterion—AIC), and each was at least moderately parameterized (had a minimum of 37 free parameters; Table 4). One might thus naïvely expect that these models, on the same data, would perform similarly. Instead, they resulted in the inference of quite divergent levels of bootstrap support for some nodes (up to a 33 percentage point difference in support; Table 4). Interestingly, the poorer-fitting models tended to find higher levels of support for both branch C of Figure 3 (a branch that was unsupported or weakly supported in earlier phylogenetic investigations [7,8,16]) and for branch L (a branch that is inconsistent with the strongly supported—74 percent ML bootstrap support and 1.0 posterior probability—results of Rothfels et al. [6]). The importance of adequate partitioning schemes for accurate phylogenetic inference has been long acknowledged [125,126], and our results mirror those of other recent empirical studies that found strong effects—both on inference of topology and support levels—of partitioning methods [122,123,127-129].

The resulting phylogenetic conclusions under our best-fitting model (model 3; see Table 4; Figure 3; Appendix S2) are comfortingly consistent with earlier results (e.g., [7,8,90]). The nine branches that are highly supported in our analyses have been inferred with high support in earlier studies, and the three branches that lack support in our data likewise have historically resisted resolution [7,8,16]. The sole exception to this pattern is the relationship among the three non-Cystopteridaceae members of the eupolypods II, which our data do not support (branch L in Figure 3), but which Rothfels et al. [6] were able to resolve with strong support (using much denser taxon sampling).

## Conclusions

The 1KP fern transcriptomes provide a powerful means to generate new single-copy nuclear regions for use by evolutionary biologists. The 20 primer pairs presented here (amplifying regions across 10 protein-coding genes) more than triple the number of such regions available for ferns. Moreover, across most of our Polypodiales genomic DNA test set (Appendix S1), the majority of these primer pairs yield PCR products that can be directly sequenced. Our sample spans the phylogenetic breadth of the Polypodiales, which includes approximately two thirds of extant fern species. Our test set, however, was focused on diploid species; researchers working with polyploids, questions of hybridization, or heterozygous individuals will need to clone their PCR products.

These newly available markers vary in their degree of variation and phylogenetic informativeness at a range of evolutionary depths (see Table 3, Figures 2, 3). In combination they yield the first broad multi-gene nuclear phylogeny for ferns. This phylogeny features strong levels of support, is consistent with the results of earlier studies, and thus provides critical evidence for the general consistency of inferences from these two genomic compartments.

For researchers working on groups outside of the Polypodiales (or those with a narrower focus within the Polypodiales), our new primers may not be directly applicable, but serve instead as a proof of concept. For these researchers, our fern-wide “all-in” alignments (see Figures S1-S9; TreeBASE accession number S14616) will provide an opportunity to design primers for their study group of choice, regardless of the position of that group within the fern phylogeny.

## Methods

### Extracting transcriptome sequences of interest and creating “all-in” alignments

**Figure 4:**
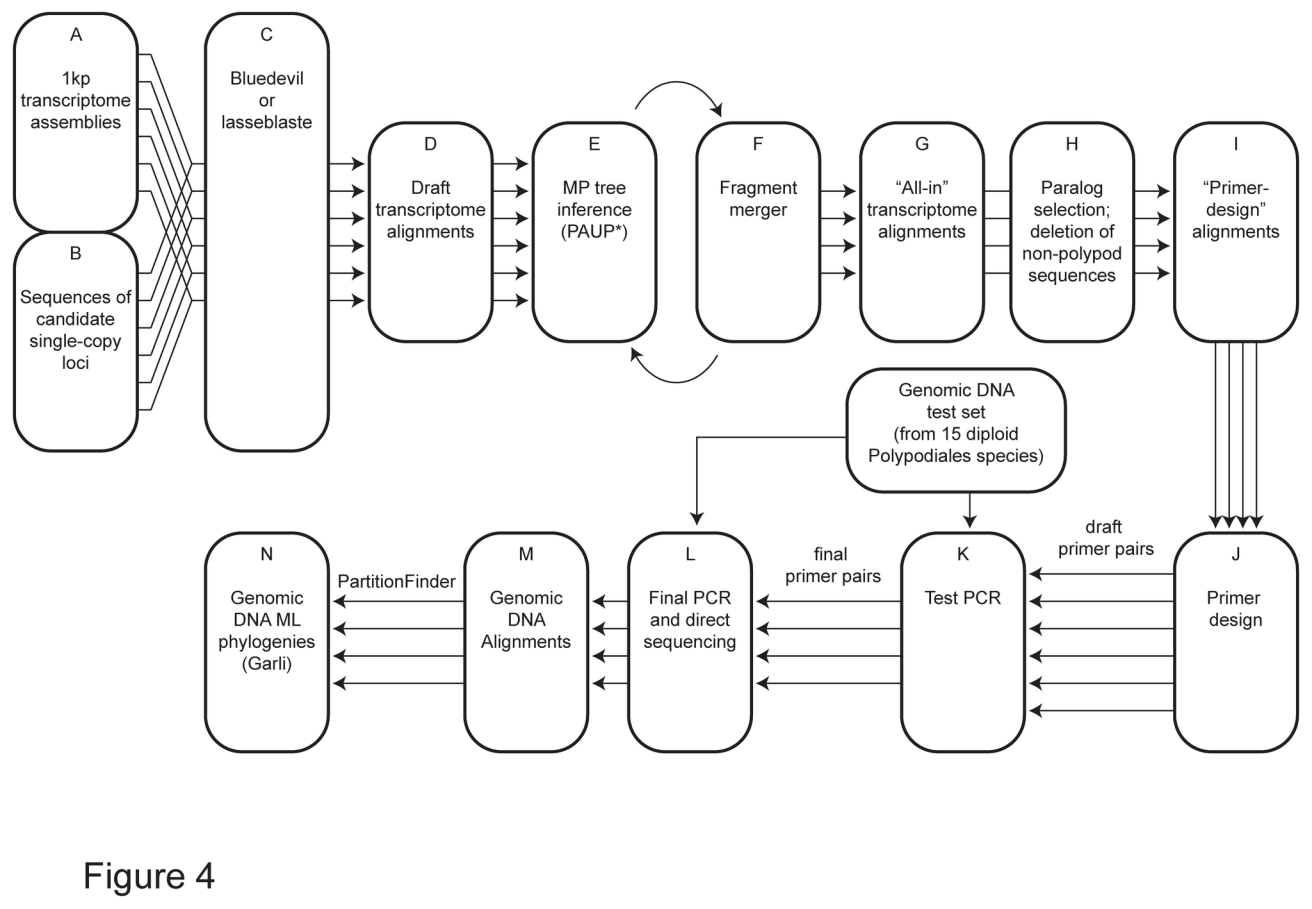
Flowchart of our transcriptome-mining pipeline.

As of January 2013, the 1KP project (www.onekp.com) had sequenced 62 fern transcriptomes, spanning the deepest branches in the fern phylogeny. RNA extraction protocols used here varied [130] although we found that the Spectrum Total Plant RNA Kit (Sigma-Aldrich, St. Louis, Missouri, U.S.A.) was effective for use with ferns. The sequencing was performed on Illumina’s GAIIx (earlier samples) or HiSeq (later samples) sequencing platforms at BGI-Shenzhen, and the 2x75 bp (GAIIx) or 2x90 bp (HiSeq) paired-end reads were assembled with *SOAPdenovo* (http://soap.genomics.org.cn/soapdenovo.html [131]) and *SOAPdenovo-trans* (http://soap.genomics.org.cn/SOAPdenovo-Trans.html); for further details on RNA extractions, transcriptome sequencing, and assembly, see Johnson et al. [130]. We took a top-down approach to finding single-copy loci in the transcriptome data. Potential single-copy loci were first selected based on personal interest or from a list of markers (generated by the 1KP project) that are putatively single-copy across a broad sample of land plants (Norman Wickett, pers. comm.). Subsequently, for each of these loci we used a combination blast [132] and tree-searching approach (Figure 4), which allowed us to confirm that the loci were single-copy (in the transcriptome data), and to focus on those with particularly good representation in the transcriptomes available to us.

We inferred fern-wide alignments for our candidate loci using one of two broad approaches (Figure 4). The first utilized the python script *Blue Devil* v0.6 [133], which detects the longest open reading frames (ORFs) in a series of query sequences, blasts those ORFs against a pool of transcriptome assemblies and provides a *MUSCLE*-based [134] alignment of the resulting hits. *Blue Devil* provides the options of using either *blastn* or *tblastx* [135], of varying the blast significance cut-off values, and of using *CAP3* [136] to re-assemble the blast hits prior to producing the alignment. *CAP3* was particularly useful in our pipeline because it allowed the *SOAPdenovo* and *SOAPdenovo-trans* assemblies of each transcriptome to be assembled together into one “master” assembly.

Our second main approach to producing transcriptome alignments was based on a nested series of blast searches using *lasseblaste* [137]. This script takes a series of query sequences as input (we used the entire pool of putatively single-copy markers listed by the 1KP project; Norman Wicket pers. comm.) and blasts each of these sequences against the pool of transcriptomes. It then takes the resulting hits and blasts them back to the full transcriptomes. From this final pool of hits, *lasseblaste* utilizes *MAFFT* [138] to produce a separate alignment of the hit sequences obtained for each query sequence and provides an accompanying quality score. The scoring system rewards alignments that have broad representation across the included transcriptomes, indicating good taxon coverage and penalizes alignments that have many hits per transcriptome, suggesting multiple paralogs and/or short read lengths. We selected five of the top 10 best-scoring of these alignments to pursue for primer design.

**Figure 5:**
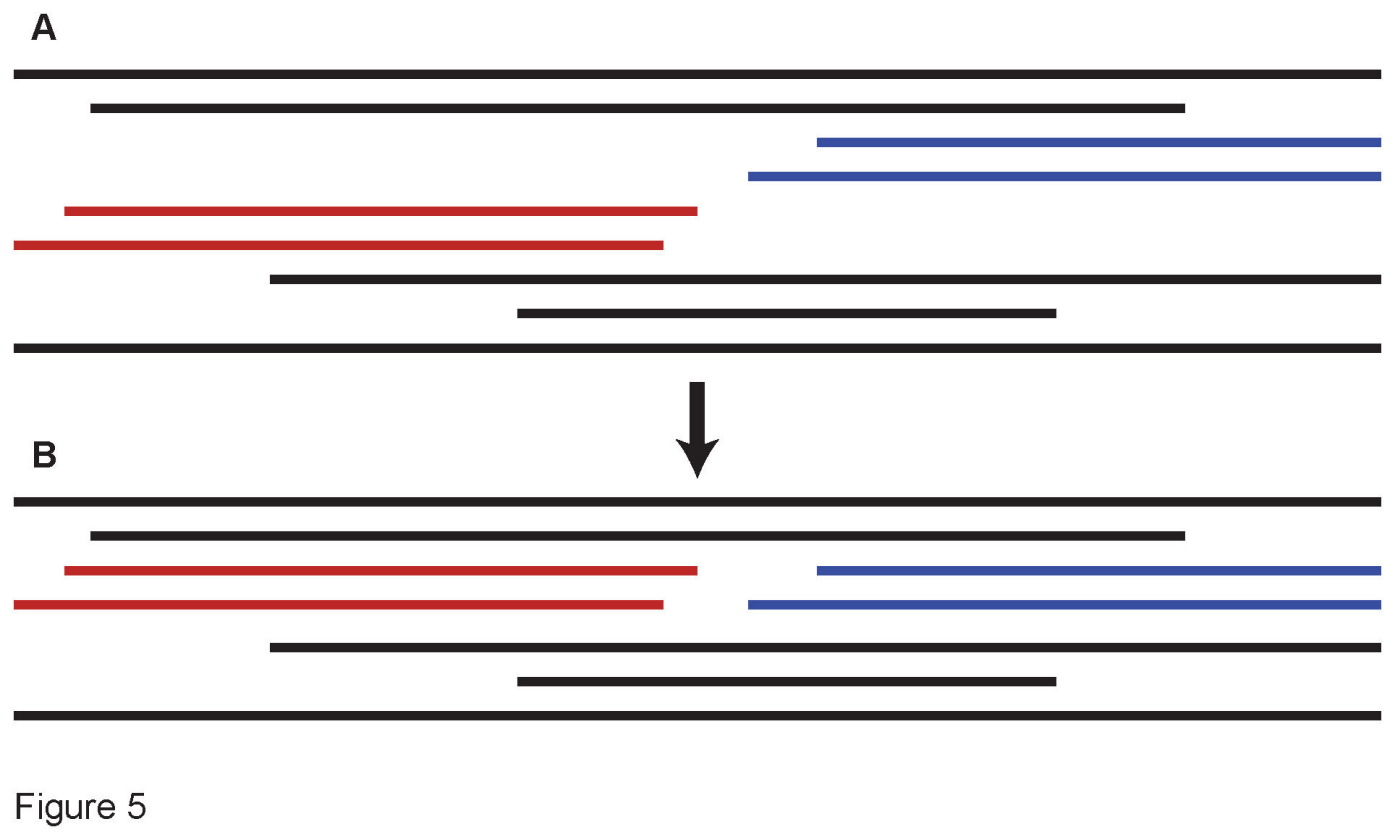
Example of our sequence-merging protocol. (A) In this schematic of a transcriptome alignment, aligned sequence fragments are indicated by the horizontal bars. Included are four fragments (colored) from our focal accession, which group together in the maximum parsimony tree. However, the two fragments from the 5’ end of the protein (in red) have some base pair conflicts with each other, as do the fragments from the 3’ end (in blue). Since the two sets of fragments do not overlap, and they group in the same area of the MP tree, it is not possible to determine which 5’ fragment belongs with which 3’ one. In this case we merged the sequences arbitrarily (B). The resulting alignment retains the full nucleotide data for primer-design purposes, but the relationships at the tips of the tree may be erroneous due to the two potentially chimaeric sequences.

Regardless of whether we used *Blue Devil* or *lasseblaste* to infer the initial alignment, we subsequently refined that alignment manually, in an iterative manner. First, we inferred a preliminary phylogenic tree from that alignment using maximum parsimony (MP) in PAUP* v4.0a125 [139]. Groups of discontinuous (or slightly overlapping) sequences from a given accession that appeared closely related in the resulting tree and did not have any conflicts with each other were combined into a single sequence in Mesquite v2.75 [140] (Figure 5). We then repeated the MP analyses on this new alignment. The resulting tree had fewer terminals, and was inferred from longer average sequences, and so provided greater power to place previously uncertain fragments. We continued this “infer-tree, group-sequences” approach until no further fragments met our criteria for merging. This process allowed us to produce an alignment with minimal missing data, and to effectively distinguish among paralogs. The final alignments generated in this way are referred to as our “all-in” alignments (see TreeBASE study number S14616).

Despite our targeting putatively single-copy genes, some of the transcriptome queries returned a variety of paralogs. In these cases, our sequence pools occasionally included two or more sequence fragments of different paralogs from a single individual taxon, where it was unclear which fragments belonged together. For example, an accession might have two fragments from the 5’ end of the protein that conflict with each other, and two conflicting sequences from the 3’ end, without any indication of which one of the 5’ sequences corresponds to which of the 3’ sequences. In this case, we created two sequences by merging the non-conflicting fragments arbitrarily (Figure 5). All sequence variation is thus preserved for primer generation purposes, but the resulting sequences may be chimeras, and their fine-scale phylogenetic relationships incorrect.

We inferred a final phylogenetic tree from each all-in alignment by ML, using *Garli* 2.0 [141], under the best-fitting model and partitioning scheme as determined by *PartitionFinder* v1.0.1 [124]. In each case we designated three data blocks (one for each codon position), and used *PartitionFinder* to evaluate all partitioning schemes, with the best selected according to the AIC. The subsequent tree searches (in *Garli*) were each run ten times, independently, from different random addition starting trees (see Figures S1-S9).

### Polypod-only alignment and primer design

From each all-in alignment we identified the copy (if multiple paralogs were present) that included the best representation of polypod sequences, and extracted those sequences to produce a new, polypod-only alignment. To this alignment we added the related *Arabidopsis thaliana* genomic DNA and cDNA sequences based on blast searches of TAIR [95] using Mesquite’s pair-wise alignment tool with a high gap-opening penalty (40). We were able to use the comparison of *Arabidopsis* genomic and cDNA sequences to estimate the location of exon-intron boundaries in the fern transcriptome sequences. In cases where the exact beginning and end of the *Arabidopsis* introns were ambiguous, we refined the boundaries to match known exon-intron boundary sequence signatures as closely as possible (e.g., see 142).

The resulting alignments are our “primer-design” alignments—they contain all available information for our taxonomic target (the Polypodiales) for each region of choice. Using the primer-design alignment we searched for conserved sites for primer design. Each primer pair was checked for hairpins, melting point, self-dimers, and hetero-dimers with Integrated DNA Technologies’ *OligoAnalyzer* v3.1 (http://www.idtdna.com/analyzer/applications/oligoanalyzer/).

### Amplification of genomic DNA and sequence characterization

Primer pairs were assayed against the test set of genomic DNA from 15 fern taxa, spanning the major polypod divergences (Appendix S1). PCR conditions followed published protocols [143] with two adjustments: (1) We incorporated one additional microliter of each primer (to compensate for primer degeneracy) and (2) reduced the volume of water by two microliters (to keep reaction size constant). Total reaction size was 21 microliters. The initial PCRs were performed across a temperature gradient, with the final optimal thermocycling conditions listed in Table 2.

For each region that amplified consistently (produced strong single bands for the majority of the test genomic DNAs), we purified and direct sequenced the products following established protocols [6,8]. For high priority targets that gave poor sequencing results, we cloned the PCR products following established protocols [64], and sequenced them as listed in Table 2. For the cryptochrome loci (*CRY2* and *CRY4*), the PCR products were gel-extracted using the QIAquick Gel Extraction Kit (QIAGEN Inc., Gaithersburg, MD) prior to cloning. We aligned the resulting sequences by hand or *MAFFT* [138] and used *Garli* v2.0 [141] to infer the best ML phylogenetic tree under a GTR+I+G model. Support was assessed via 1000 bootstrap pseudoreplicates, with each bootstrap tree search performed twice, from different random addition starting trees (Figure 2).

Due to the breadth of our taxon sample, much of the intron data could not be unambiguously aligned and thus were excluded prior to tree-searching, which reduced our ability to assess the utility of these markers at shallower phylogenetic depths. To overcome this weakness, we chose two pairs of closely related species (*Cystopteris bulbifera* and *C. protrusa* and *Polypodium amorphum* and *P. glycyrrhiza*) to provide metrics for the variability of each region. For each species pair we computed the total number of base differences between the sequences of the two species (with each indel counted as a single “difference” regardless of its length) for each region (Table 3). All newly generated genomic sequences are available in GenBank (Appendix S1).

### Polypodiales combined data phylogeny

To demonstrate the utility of our markers across various phylogenetic depths (the earliest divergences in the Polypodiales occurred approximately 190 million years ago [144]) and to attempt to resolve polytomies in the backbone of the Polypodiales phylogeny [8,20] we combined the genomic DNA alignments for our loci and inferred their phylogeny by ML. Some of the locus alignments contained multiple sequences for individual accessions (representing paralogs, or allelic variation; see Figure 2). In these cases, the longest sequence was retained. In the event of a predicted duplication affecting multiple accessions, the copy that had the greatest average length was kept, rather than the longest sequence within each copy. The resulting alignments were combined into a single alignment using *abioscripts* (available at http://ormbunkar.se/phylogeny/abioscripts/). This script produces a concatenated alignment, inserting blank characters for accessions not represented in a particular locus, while maintaining exclusion set, codon position, and character set information.

We used *PartitionFinder* v1.0.1 [124] to find the best model for the analysis of these data. We performed three *PartitionFinder* runs to investigate a spectrum of possible models (Table 4). The first had four predefined data blocks (one for each codon position, and one for the noncoding sequences), the second had 19 data blocks (one for each locus), and the third had 72 data blocks (each codon position/noncoding sequence considered separately, for each locus). For each of these three runs, we set *PartitionFinder* to find the best partitioning scheme while considering all possible substitution models (with subset-specific substitution models selected by the AIC), testing all possible schemes in the first case, and using a greedy heuristic for the latter two runs. We selected the final model (optimal partitioning scheme with accompanying substitution models for each subset) by fit, as assessed by the BIC. To this set of three models, we added two others (see Table 4). The simplest is an unpartitioned GTR+I+G model, and the more complicated is partitioned by locus, with each locus given its own best-fit substitution model (manually derived from the subset output files from the by-locus *PartitionFinder* run).

We performed ML tree searches under these five models in *Garli* 2.0 [141]. For each model we did 10 best-tree searches, from different random-addition sequence starting trees, and assessed support via 1000 bootstrap pseudoreplicates, each from a single random-addition starting tree (Table 4, Figure 3). These bootstrap runs, and other computation-intensive analyses, were run on the Duke Shared Cluster Resource (https://wiki.duke.edu/display/SCSC/DSCR).

## Supporting Information

Appendix S1 **Voucher data and GenBank accession numbers for our Polypodiales genomic DNA test set.** Numbers in parenthesis following the species names are Fern Lab Database accession numbers (fernlab.biology.duke.edu); letters in parentheses are acronyms for the herbaria where the vouchers are deposited, from Index Herbariorum [145]. Missing data are indicated by an n-dash (“-”).
(DOCX)

Appendix S2 **Full description of partitioning schemes and substitution models applied for the five models investigated (1, 2a, 2b, 3, and 4).** In the "Subset Contents" field for model 2a, terminal digits refer to codon position: _1= First codon position; _2= Second codon position; _3= Third codon position; _N= Non-coding sequence.
(XLSX)

Figure S1 ***ApPEFP* all-in maximum likelihood transcriptome phylogeny.**
(PDF)

Figure S2 ***CRY* all-in maximum likelihood transcriptome phylogeny.** a) preduplication *CRY3/4*, *CRY3*, and *CRY4*; b) *CRY5*, preduplication *CRY1/2*, and *CRY2*; c) *CRY1*, and a cartoon “map” of the entire cryptochrome fern phylogeny.
(PDF)

Figure S3 ***DET1* all-in maximum likelihood transcriptome phylogeny.**
(PDF)

Figure S4 ***GAP* all-in maximum likelihood transcriptome phylogeny.** a) *gapA, gapB*, mystery *gap*, and *gapC*; b) *gapCp* (including *Cp* *Short* and *Cp* *Long*), and a cartoon map of the *GAP* family phylogeny.
(PDF)

Figure S5 ***IBR3* all-in maximum likelihood transcriptome phylogeny.**
(PDF)

Figure S6 ***pgiC* all-in maximum likelihood transcriptome phylogeny.**
(PDF)

Figure S7 ***SDQ1* all-in maximum likelihood transcriptome phylogeny.**
(PDF)

Figure S8 ***TPLATE* all-in maximum likelihood transcriptome phylogeny.**
(PDF)

Figure S9 ***transducin* all-in maximum likelihood transcriptome phylogeny.**
(PDF)
