# Supplementary material for: Transcriptome-Mining for Single-Copy Nuclear Markers in Ferns
Source: PLoS One. 2013 Oct 8;8(10):e76957. doi: 10.1371/journal.pone.0076957 (PMC3792871; doi:10.1371/journal.pone.0076957)
Supplement: Appendix S1 — Voucher data and GenBank accession numbers for our Polypodiales genomic DNA test set. Numbers in parenthesis following the species names are Fern Lab Database accession numbers (fernlab.biology.duke.edu); letters in parentheses are acronyms for the herbaria where the vouchers are deposited, from Index Herbariorum [145]. Missing data are indicated by an n-dash (“-”). (DOCX) [file pone.0076957.s001.docx]

# Appendix S1

Voucher data and GenBank accession numbers for our Polypodiales genomic DNA test set. GenBank numbers are presented in the following order: *ApPEFP_C* Region1, *ApPEFP_C* Region1a, *ApPEFP_C* Region1b, *ApPEFP_C* Region2, *ApPEFP_C* Region3, *CRY2* Region1, *CRY4* Region1, *DET1* Region1, *gapCpSh* Region1, *IBR3* Region1, *IBR3* Region2, *pgiC* Region1, *SQD1* Region1, *SQD1* Region1a, *SQD1* Region2, *TPLATE* Region1, TPLATE Region2, *transducin* Region1, *transducin* Region2, *transducin* Region3. Numbers in parenthesis following the species names are Fern Lab Database accession numbers (fernlab.biology.duke.edu); letters in parentheses are acronyms for the herbaria where the vouchers are deposited, from Index Herbariorum [145]. Missing data are indicated by an n-dash (“–”).

**CYATHEALES**: *Alsophila podophylla* Hook. (4948). Schuettpelz et al. 1201A (DUKE). Taiwan: Ilan Co. –; ‡; –; KF553697; –; KF553754 and KF553755; –; –; KF553792; –; KF553739; –; –; KF553813; KF553828; –; –; –; –; –. *Dicksonia sellowiana* Hook. (–). Larsson 804 (UPS). Brazil: Cult Uppsala Bot. Garden. –; –; –; –; –; –; –; –; –; –; –; –; –; –; –; KF553848; –; –; KF553879; –. **LINDSAEACEAE**: *Lindsaea lancea* (L.) Bedd. (7372). Rothfels et al. 3616 (DUKE). Ecuador: Zamora-Chinchipe. –; –; –; KF553705; KF553717; KF553763; KF553777; –; KF553799; –; KF553747; –; KF553821; –; KF553836; KF553850; –; –; –; –. **SACCOLOMATACEAE**: *Saccoloma inaequale* (Kunze) Mett. (5106). Sundue 1360 (NY). Costa Rica: Heredia. KF553679; –; –; KF553706; –; KF553766; KF553779; –; KF553802; KF553735; KF553750; –; KF553824; –; KF553839; –; –; –; –; –. **PTERIDACEAE**: *Adiantum aleuticum* (Rupr.) C.A. Paris (8577). Rothfels & Zylinski 4097 (DUKE). Canada: British Columbia. –; –; –; –; –; –; –; KF553781; –; –; –; –; –; –; –; –; –; –; –; –. *Adiantum pedatum* L. (8974). Rothfels & Rushworth 4166 (DUKE). U.S.A.: North Carolina. KF553667; –; KF553691; KF553696; KF553709; KF553753; KF553769; –; KF553791; KF553723; KF553738; –; KF553812; –; KF553827; KF553841; –; KF553864; –; KF553885. *Cheilanthes covillei* Maxon (3845). Windham & Pryer 3436 (DUKE). U.S.A.: California. KF553670; KF553683; –; KF553700; KF553711; KF553758; KF553771; –; KF553793; KF553725; KF553741; –; KF553815; –; KF553830; KF553843; –; KF553866; KF553875; KF553887. *Cryptogramma acrostichoides* R.Br. (8514). Rothfels & Zylinski 4078 (DUKE). Canada: British Columbia. –; –; –; –; –; –; –; –; –; –; –; –; –; –; –; KF553844; –; KF553867; –; KF553888. *Cryptogramma acrostichoides* R.Br. (8525). Rothfels & Zylinski 4088.1 (DUKE). Canada: British Columbia. KF553671; –; –; KF553701; KF553712; KF553759; –; –; KF553794; KF553726; KF553742; –; KF553816; –; KF553831; –; –; –; –; –. **DENNSTAEDTIACEAE**: *Dennstaedtia punctilobula* (Michx.) T.Moore (8975). Rothfels & Rushworth 4167 (DUKE). U.S.A.: North Carolina. KF553673; KF553685; KF553693; KF553703; KF553715; –; KF553775; –; KF553797; KF553729; KF553745; –; KF553819; –; KF553834; KF553847; KF553859; KF553870; KF553878; –. **EUPOLYPODS I**: *Dryopteris intermedia* (Muhl. ex Willd.) A.Gray (8720). Tripp 224 (DUKE). U.S.A.: North Carolina. –; –; –; –; –; KF553762; –; –; –; –; –; –; –; –; –; –; –; –; –; –. *Dryopteris intermedia* (Muhl. ex Willd.) A.Gray (8971). Rothfels & Rushworth 4163 (DUKE). U.S.A.: North Carolina. KF553674; KF553686; KF553694; KF553704; KF553716; –; KF553776; KF553785; KF553798; KF553730 and KF553731; KF553746; KF553808; KF553820; –; KF553835; KF553849; KF553860; –; KF553880; KF553891. *Polypodium amorphum* Suksd. (7771). Sigel 2010-125 (DUKE). U.S.A.: Washington. KF553675 and KF553676; KF553688; –; –; KF553718; KF553764; –; KF553786; KF553800; KF553732 and KF553733; KF553748; KF553809; –; KF553822; KF553837; KF553851; KF553861; KF553871; KF553881; KF553892. *Polypodium glycyrrhiza* D.C. Eaton (8523). Rothfels & Zylinski 4086 (DUKE). Canada: British Columbia. KF553677 and KF553678; KF553687; ‡; –; KF553719 and KF553720; KF553765; KF553778; KF553787; KF553801; KF553734; KF553749; KF553810; KF553823; –; KF553838; KF553852; KF553862; –; KF553882; KF553893. **EUPOLYPODS II**: *Athyrium filix-femina* (L.) Roth (8973). Rothfels & Rushworth 4165 (DUKE). U.S.A.: North Carolina. KF553668; KF553682; KF553692; KF553698; KF553710; KF553756 and KF553757; KF553770; KF553782; –; KF553724; KF553740; KF553805; KF553814; –; KF553829; KF553842; KF553856; KF553865; KF553874; KF553886. *Cystopteris bulbifera* (L.) Bernh. (7667). Rothfels & Rothfels 3947 (DUKE). Canada: Ontario. KF553669; KF553684; –; KF553699; KF553713; KF553760; KF553772 and KF553773; KF553783; KF553795; KF553727; KF553743; KF553806; KF553817; –; KF553832; KF553845; KF553857; KF553868; KF553876; KF553889. *Cystopteris protrusa* (Weath.) Blasdell (6454). Rothfels 2890 (DUKE). U.S.A.: Virginia. KF553666 and KF553672; ‡; –; KF553702; KF553714; KF553761; KF553774; KF553784; KF553796; KF553728; KF553744; KF553807; KF553818; –; KF553833; KF553846; KF553858; KF553869; KF553877; KF553890. *Thelypteris noveboracensis* (L.) Nieuwl. (8972). Rothfels & Rushworth 4164 (DUKE). U.S.A.: North Carolina. KF553680; KF553689; KF553695; KF553707; KF553721; KF553767; KF553780; KF553788; KF553803; KF553736; KF553751; KF553811; KF553825; –; KF553840; KF553853; KF553855; KF553872; KF553883; KF553894. *Woodsia ilvensis* (L.) R.Br. (7968). Larsson 303 (UPS). Norway: Troms. KF553681; KF553690; –; KF553708; KF553722; KF553768; –; KF553789; KF553790; KF553737; KF553752; KF553804; KF553826; –; –; KF553854; KF553863; KF553873; KF553884; –.

‡ These sequences were less that 200 basepairs long, and were thus not accepted for archiving by GenBank. They are available from CJR by request.
