# Supplementary figures and images for: Transcriptome-Mining for Single-Copy Nuclear Markers in Ferns
Source: PLoS One. 2013 Oct 8;8(10):e76957. doi: 10.1371/journal.pone.0076957 (PMC3792871; doi:10.1371/journal.pone.0076957)

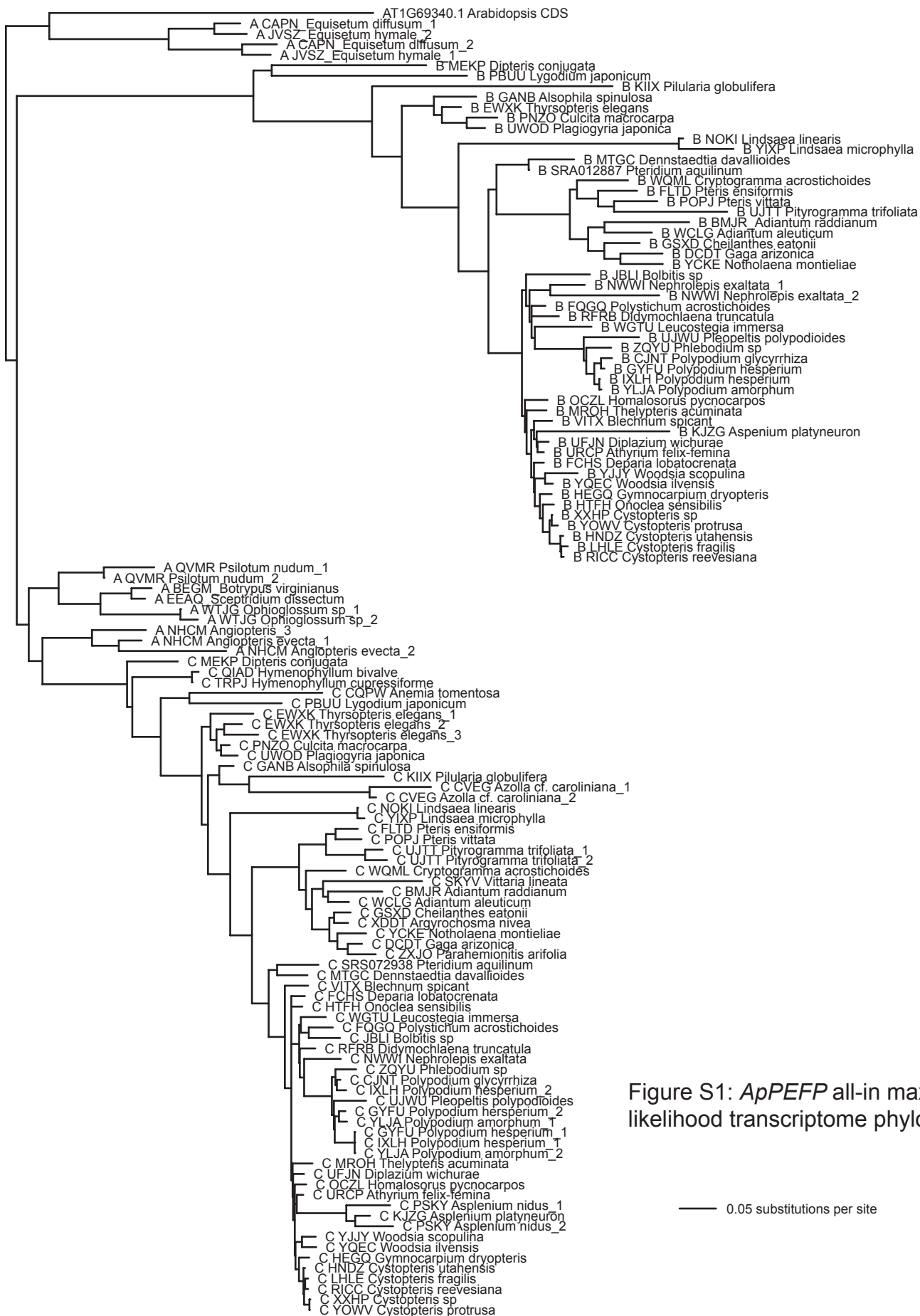

Figure S1: *ApPEFP* all-in maximum likelihood transcriptome phylogeny

Supplement: Figure S1 — ApPEFP all-in maximum likelihood transcriptome phylogeny. (PDF) [file pone.0076957.s003.pdf]

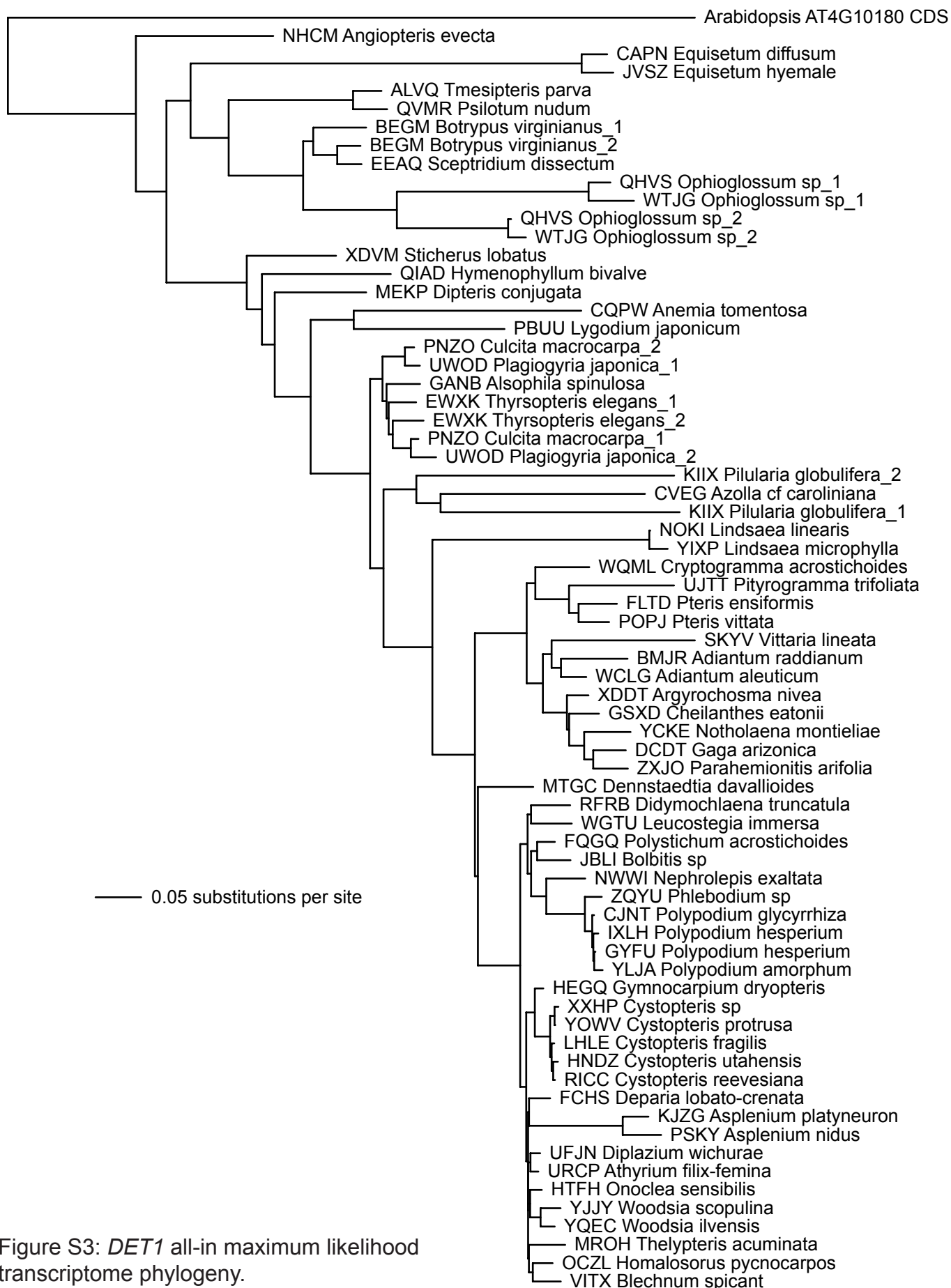

Figure S3: *DET1* all-in maximum likelihood transcriptome phylogeny.

Supplement: Figure S3 — DET1 all-in maximum likelihood transcriptome phylogeny. (PDF) [file pone.0076957.s005.pdf]

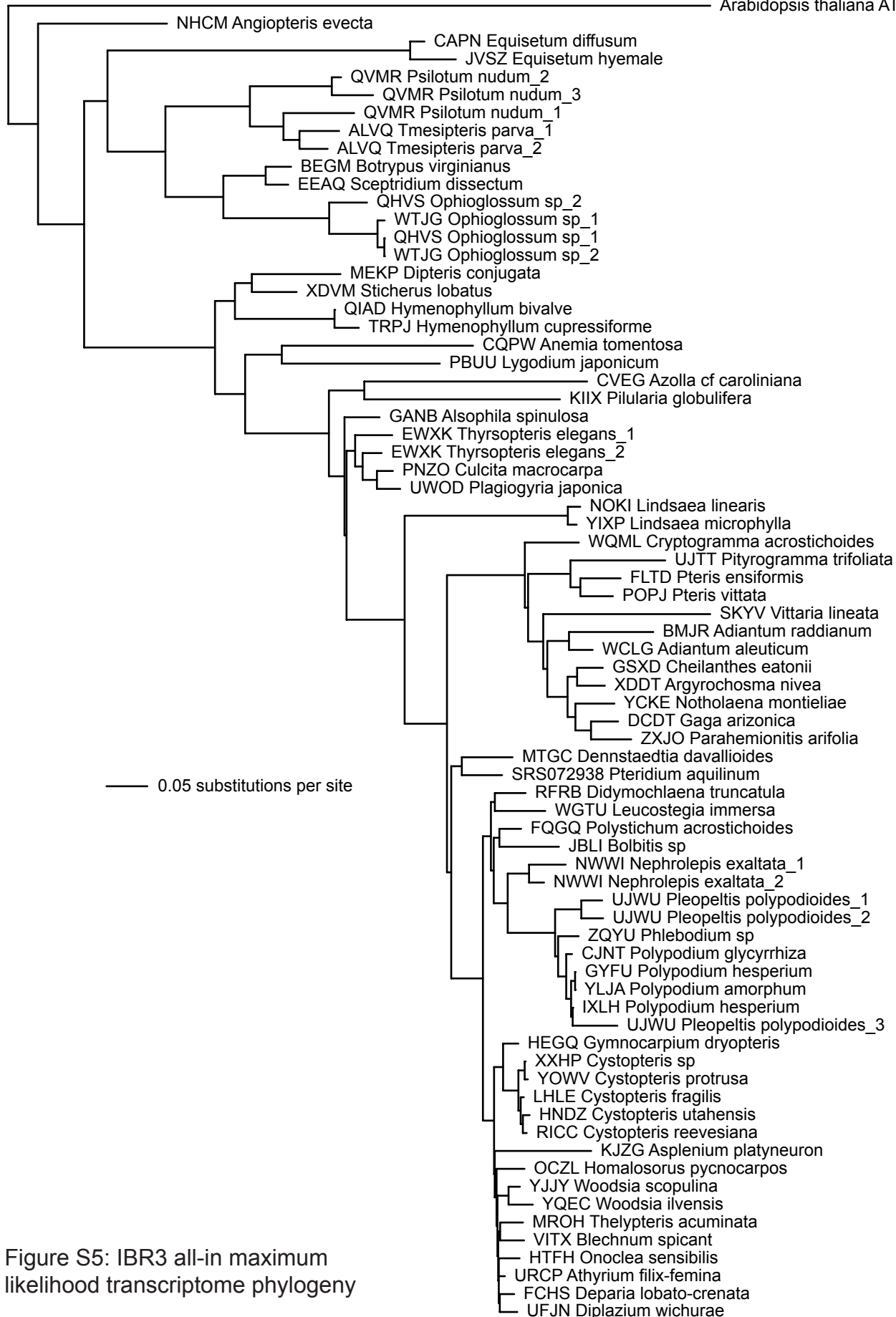

Figure S5: IBR3 all-in maximum likelihood transcriptome phylogeny

Supplement: Figure S5 — IBR3 all-in maximum likelihood transcriptome phylogeny. (PDF) [file pone.0076957.s007.pdf]

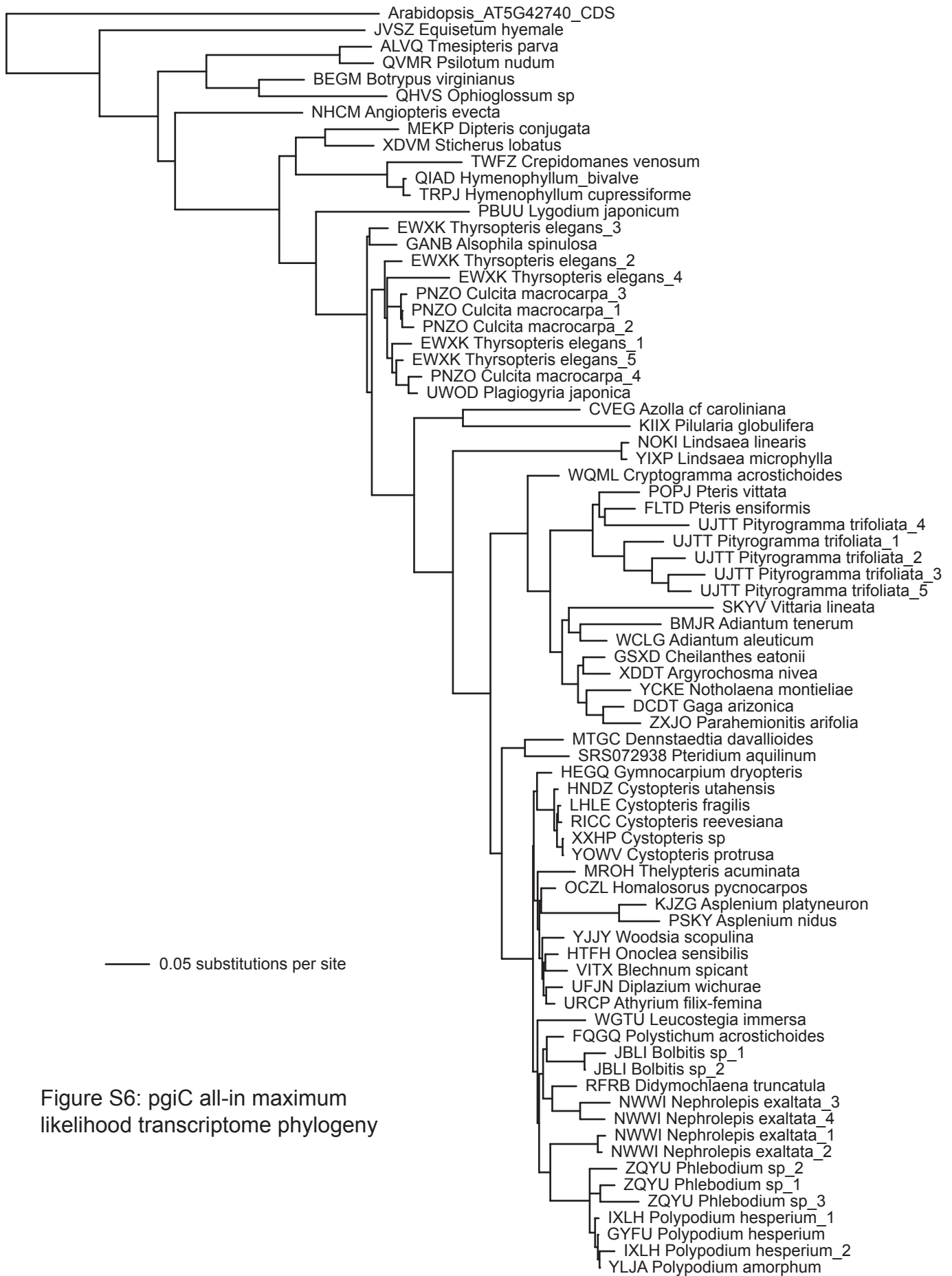

Figure S6: *pgc* all-in maximum likelihood transcriptome phylogeny

Supplement: Figure S6 — pgiC all-in maximum likelihood transcriptome phylogeny. (PDF) [file pone.0076957.s008.pdf]

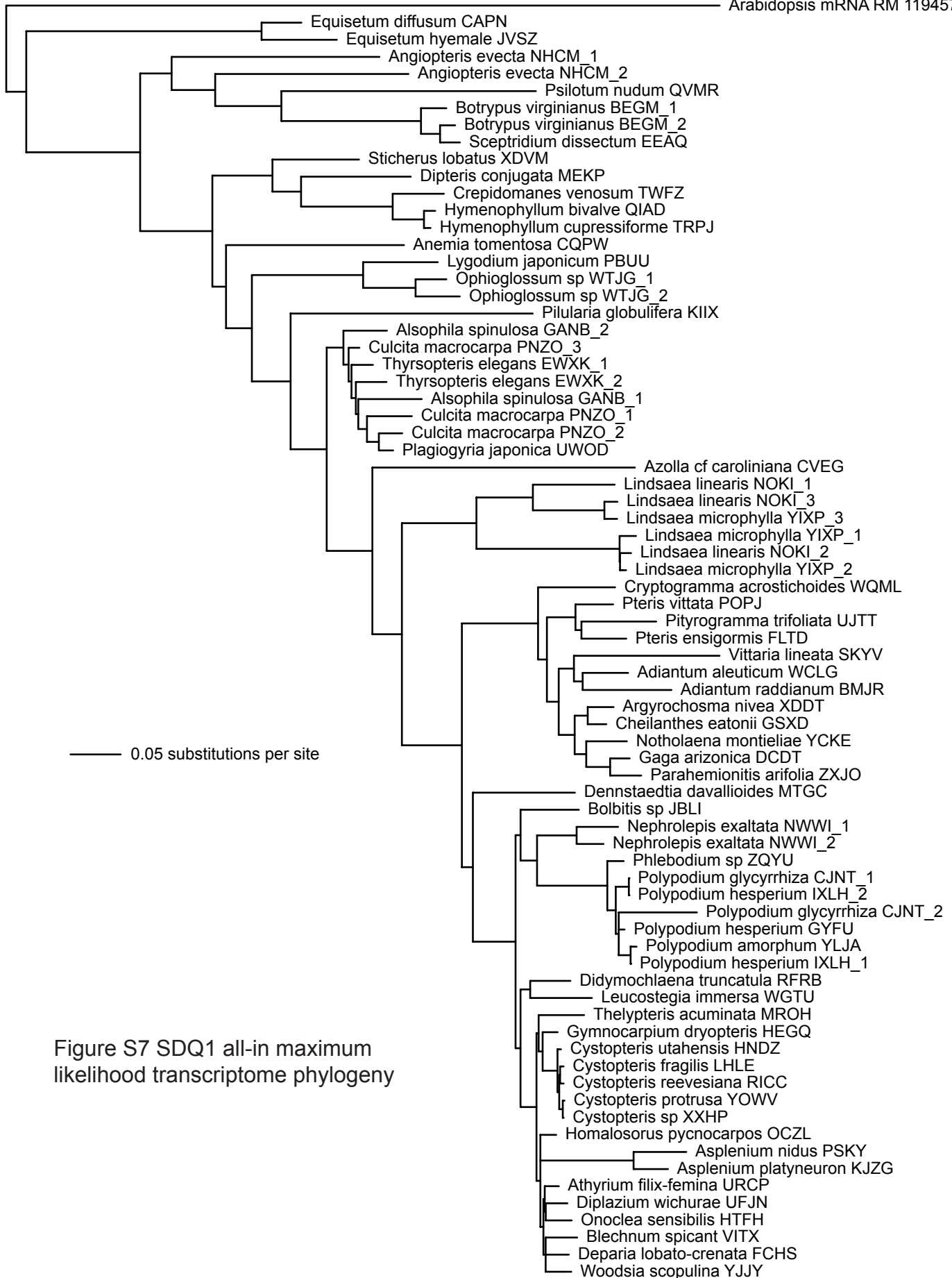

Figure S7 SDQ1 all-in maximum likelihood transcriptome phylogeny

Supplement: Figure S7 — SDQ1 all-in maximum likelihood transcriptome phylogeny. (PDF) [file pone.0076957.s009.pdf]

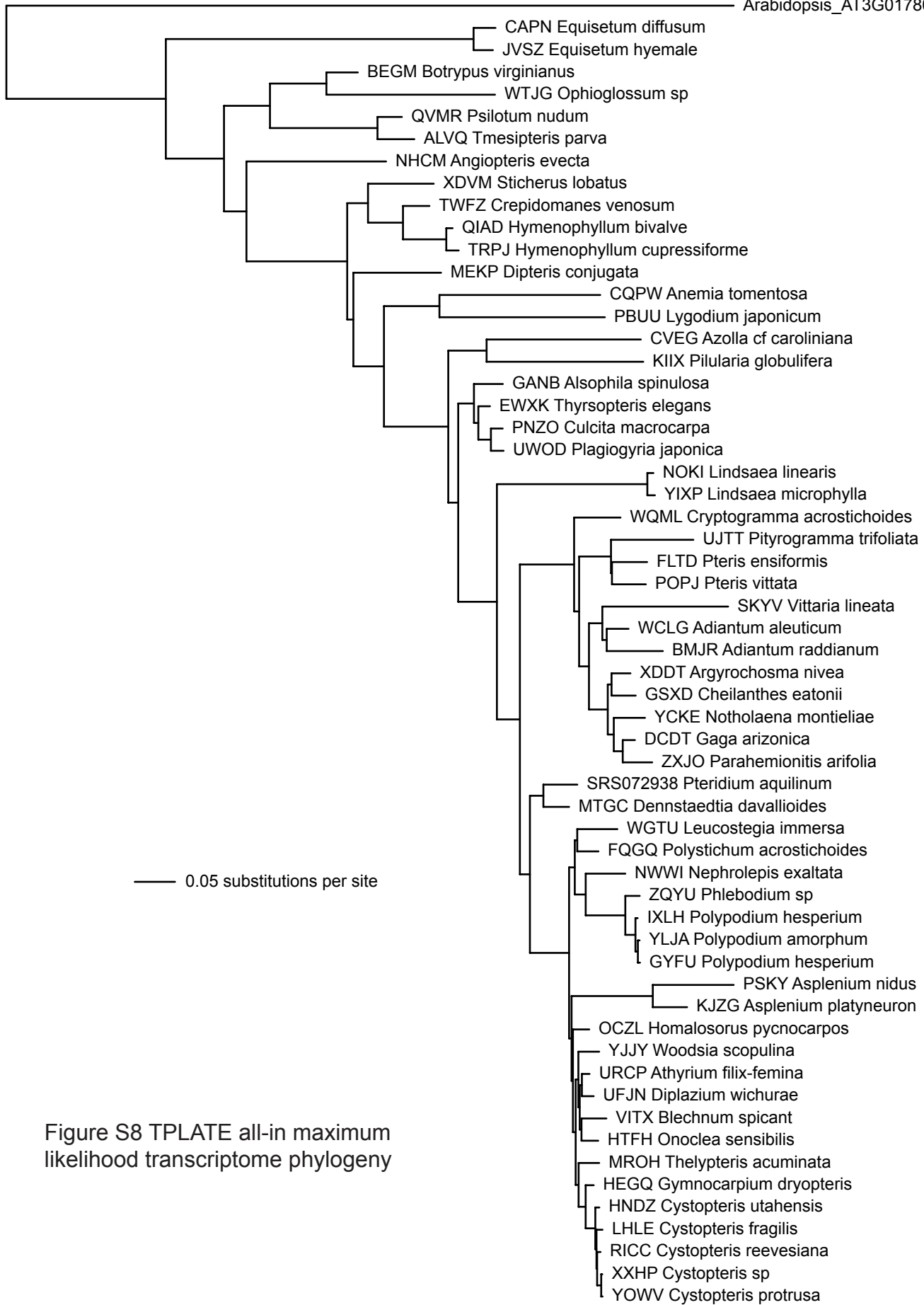

Figure S8 TPLATE all-in maximum likelihood transcriptome phylogeny

Supplement: Figure S8 — TPLATE all-in maximum likelihood transcriptome phylogeny. (PDF) [file pone.0076957.s010.pdf]

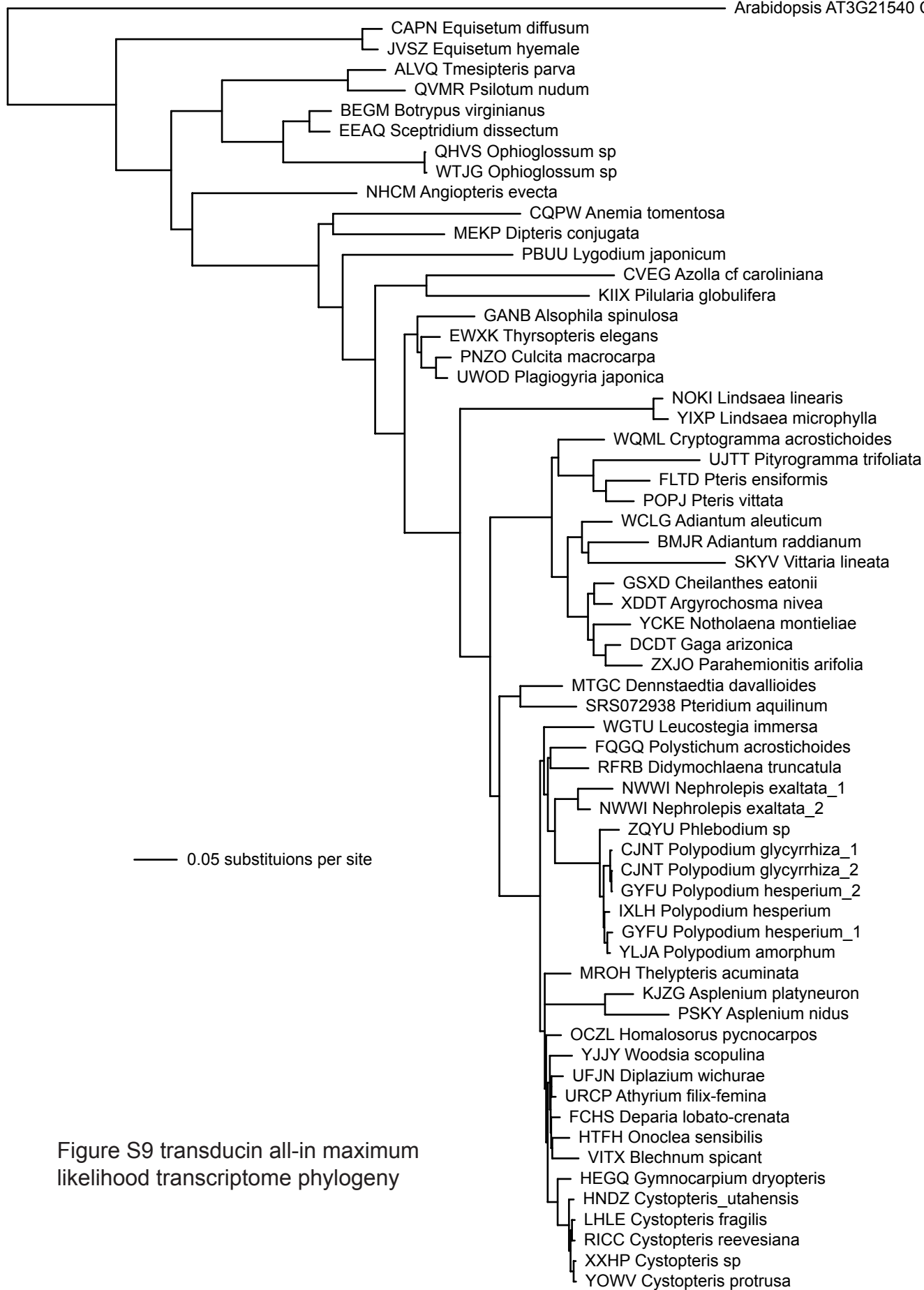

Figure S9 transducin all-in maximum likelihood transcriptome phylogeny

Supplement: Figure S9 — transducin all-in maximum likelihood transcriptome phylogeny. (PDF) [file pone.0076957.s011.pdf]
