# Supplementary material for: Transcriptome-Mining for Single-Copy Nuclear Markers in Ferns
Source: PLoS One. 2013 Oct 8;8(10):e76957. doi: 10.1371/journal.pone.0076957 (PMC3792871; doi:10.1371/journal.pone.0076957)
Supplement: Figure S2 — CRY all-in maximum likelihood transcriptome phylogeny. a) preduplication CRY3/4, CRY3, and CRY4; b) CRY5, preduplication CRY1/2, and CRY2; c) CRY1, and a cartoon “map” of the entire cryptochrome fern phylogeny. (PDF) [file pone.0076957.s004.pdf]

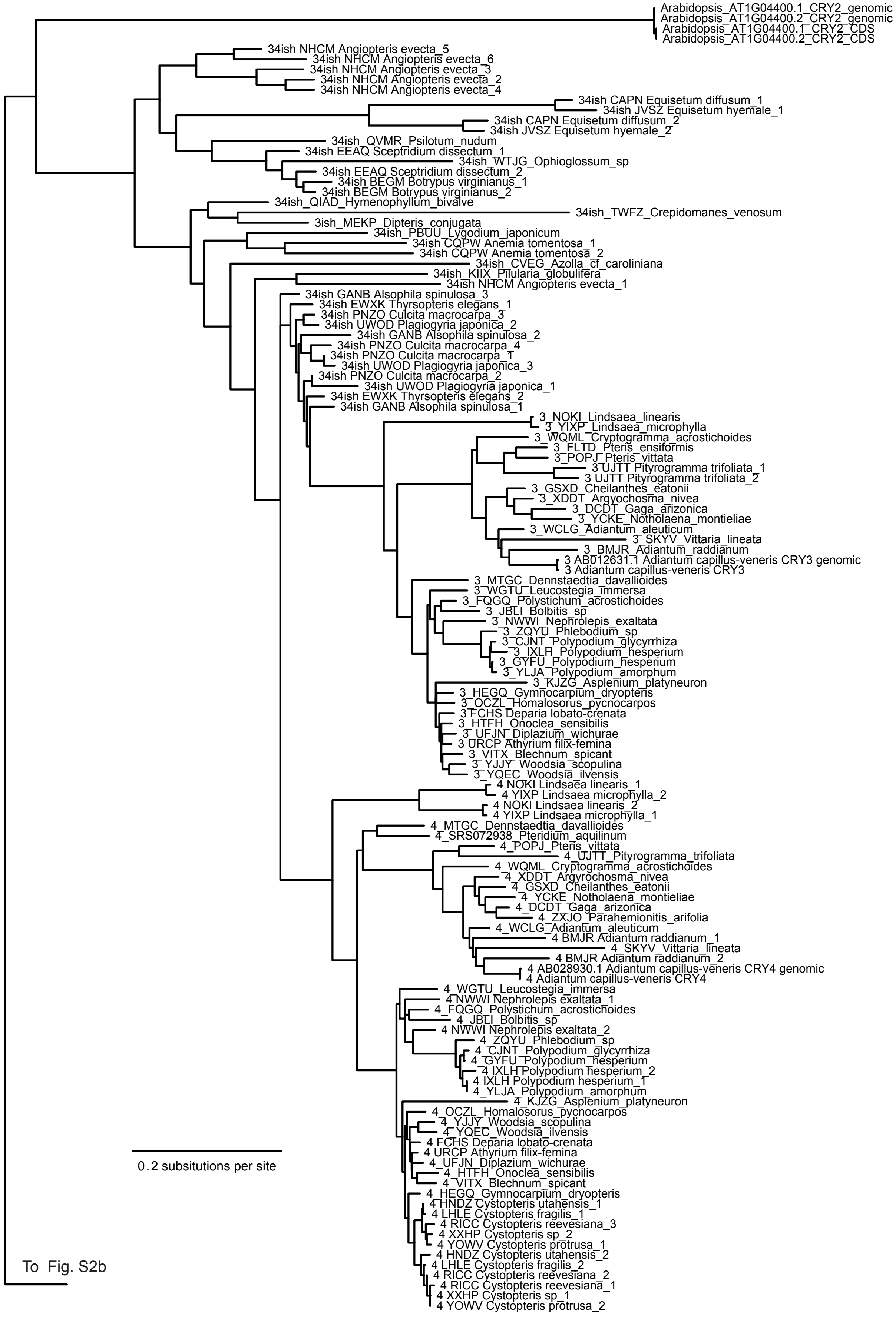

Figure S2a: CRY all-in maximum likelihood transcriptome phylogeny: preduplication CRY3/4, CRY3, and CRY4.

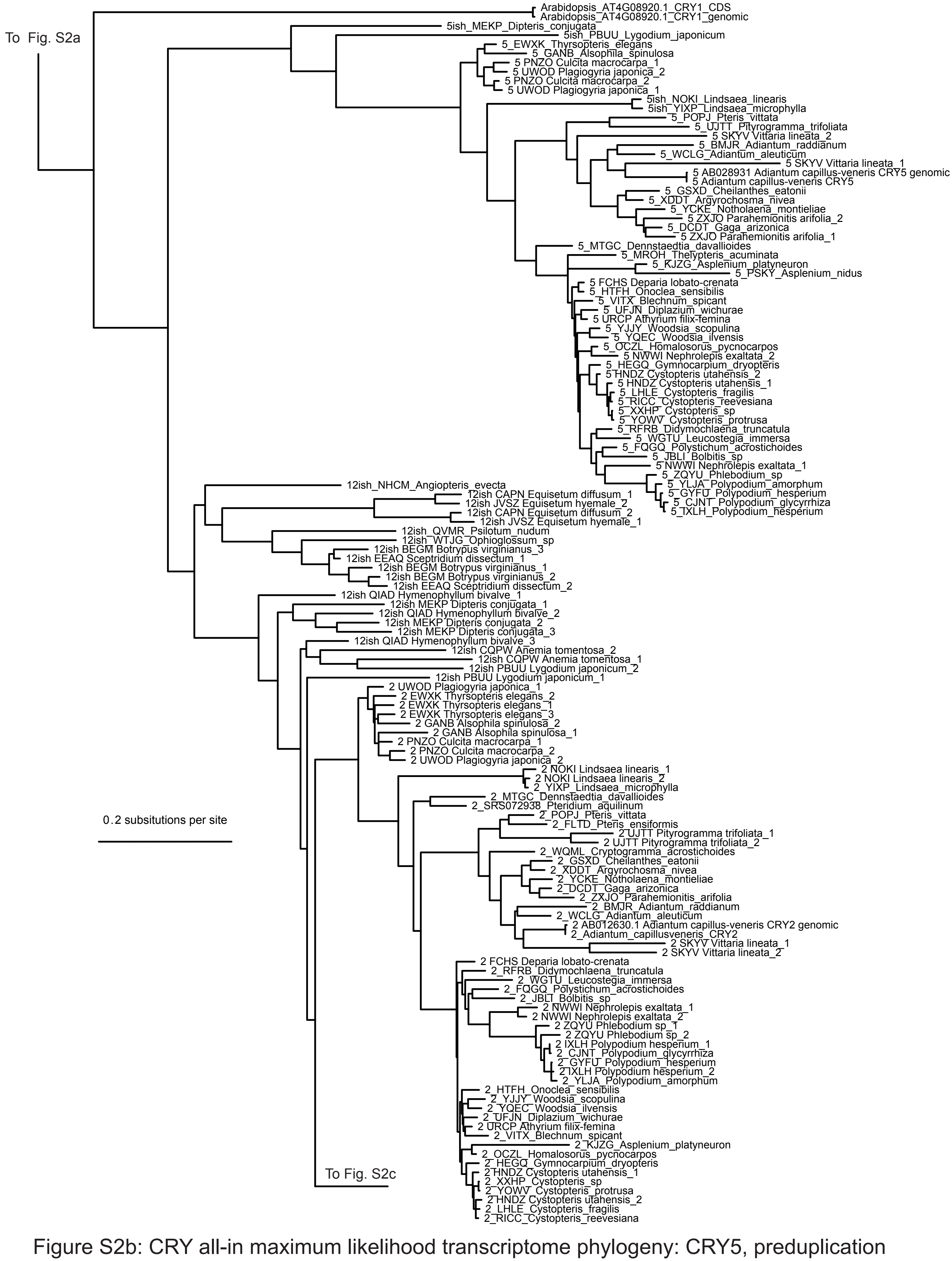

Figure S2b: CRY all-in maximum likelihood transcriptome phylogeny: CRY5, preduplication CRY1/2, and CRY2.

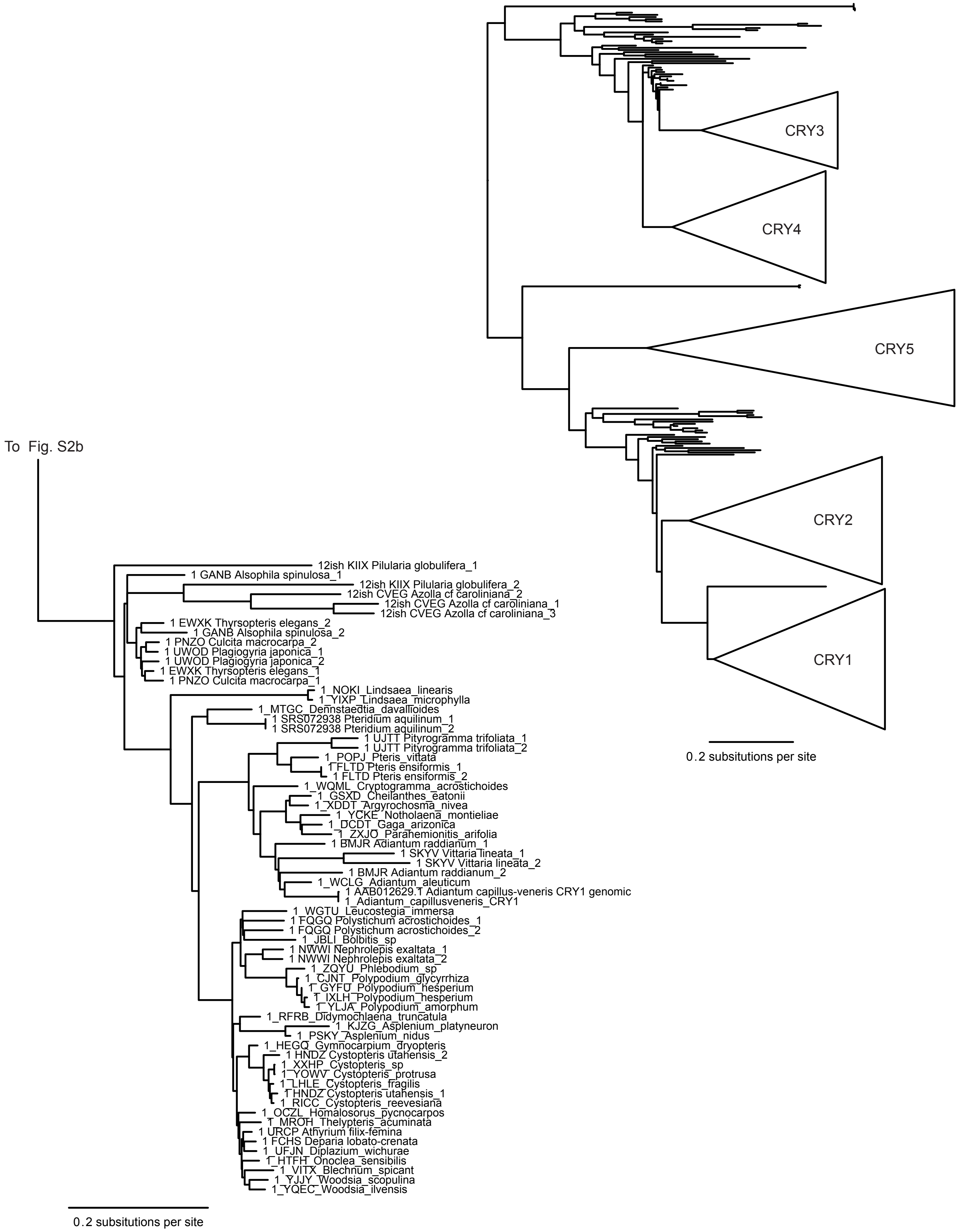

Figure S2c: CRY all-in maximum likelihood transcriptome phylogeny: CRY1, and a cartoon "map" of the entire cryptochrome fern phylogeny.
