# Supplementary material for: Transcriptome-Mining for Single-Copy Nuclear Markers in Ferns
Source: PLoS One. 2013 Oct 8;8(10):e76957. doi: 10.1371/journal.pone.0076957 (PMC3792871; doi:10.1371/journal.pone.0076957)
Supplement: Figure S4 — GAP all-in maximum likelihood transcriptome phylogeny. a) gapA, gapB, mystery gap, and gapC; b) gapCp (including Cp Short and Cp Long), and a cartoon map of the GAP family phylogeny. (PDF) [file pone.0076957.s006.pdf]

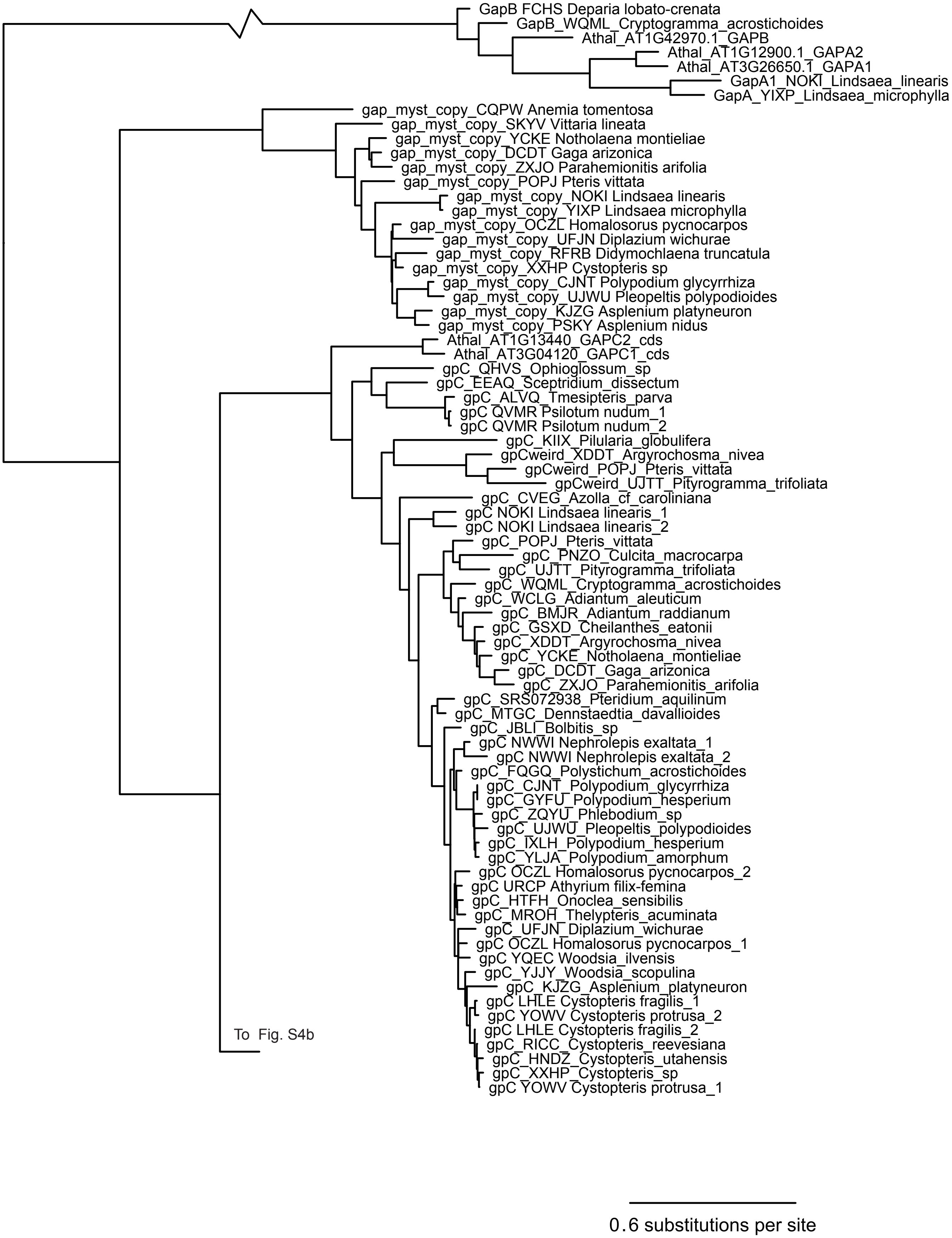

Figure S4a: GAP all-in maximum likelihood transcriptome phylogeny: gapA, gapB, mystery gap, and gapC.

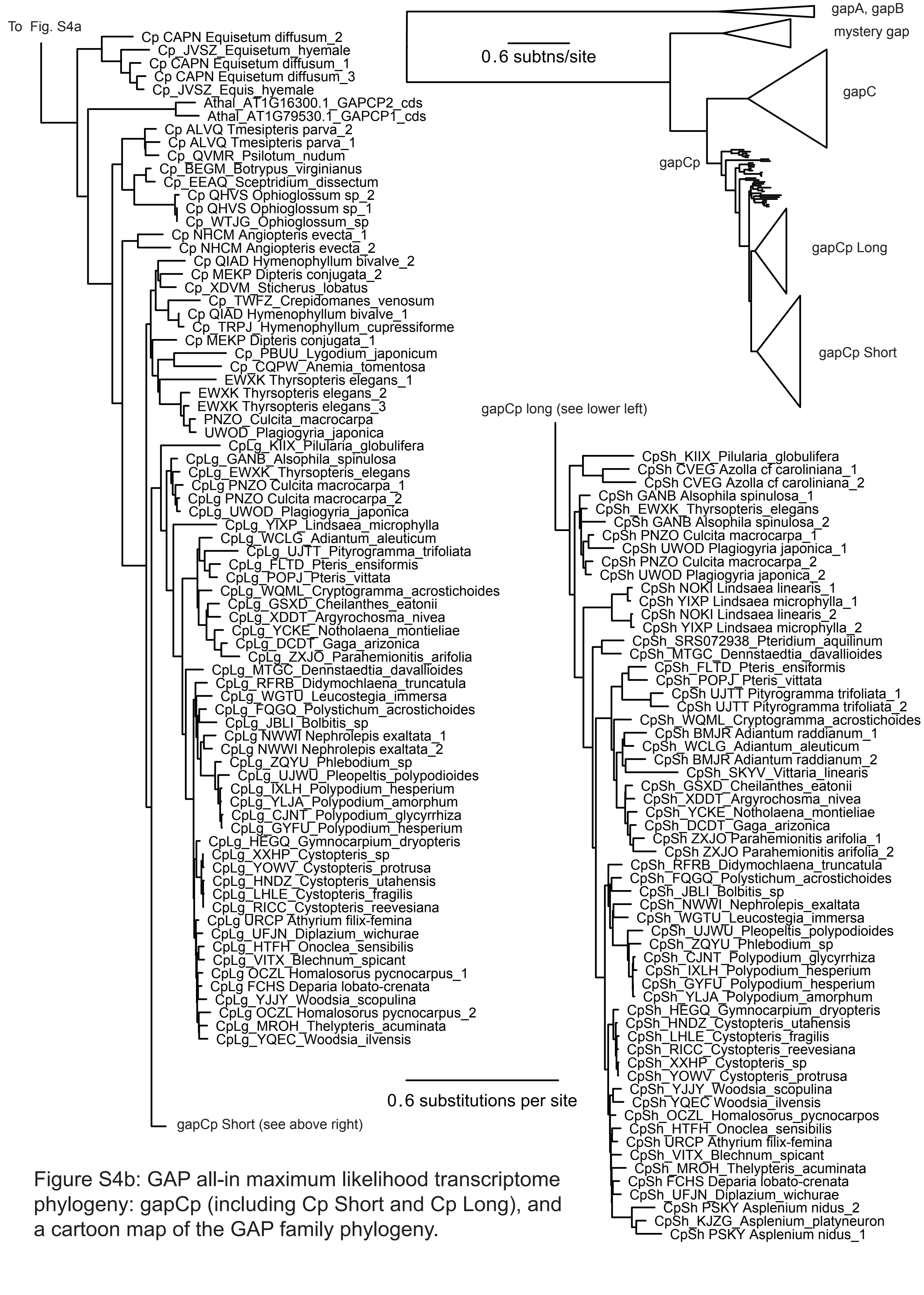

Figure S4b: GAP all-in maximum likelihood transcriptome phylogeny: gapCp (including Cp Short and Cp Long), and a cartoon map of the GAP family phylogeny.
